# Discovery and Folding Dynamics of a Fused Bicyclic Cysteine Knot Undecapeptide from the Marine Sponge *Halichondria bowerbanki*

**DOI:** 10.1021/acs.joc.4c01104

**Published:** 2024-08-27

**Authors:** Weimao Zhong, Jeremiah O. Olugbami, Prashanth Rathakrishnan, Ipsita Mohanty, Samuel G. Moore, Neha Garg, Adegboyega K. Oyelere, Thomas L. Turner, Andrew C. McShan, Vinayak Agarwal

**Affiliations:** † School of Chemistry and Biochemistry, Georgia Institute of Technology, Atlanta, Georgia 30332, United States; ‡ Department of Biochemistry, University of Ibadan, Ibadan, Oyo 200005, Nigeria; § Petit Institute for Bioengineering and Bioscience, Georgia Institute of Technology, Atlanta, Georgia 30332, United States; ∥ Ecology, Evolution, and Marine Biology Department, University of California Santa Barbara, Santa Barbara, California 93106, United States; ⊥ School of Biological Sciences, Georgia Institute of Technology, Atlanta, Georgia 30332, United States

## Abstract

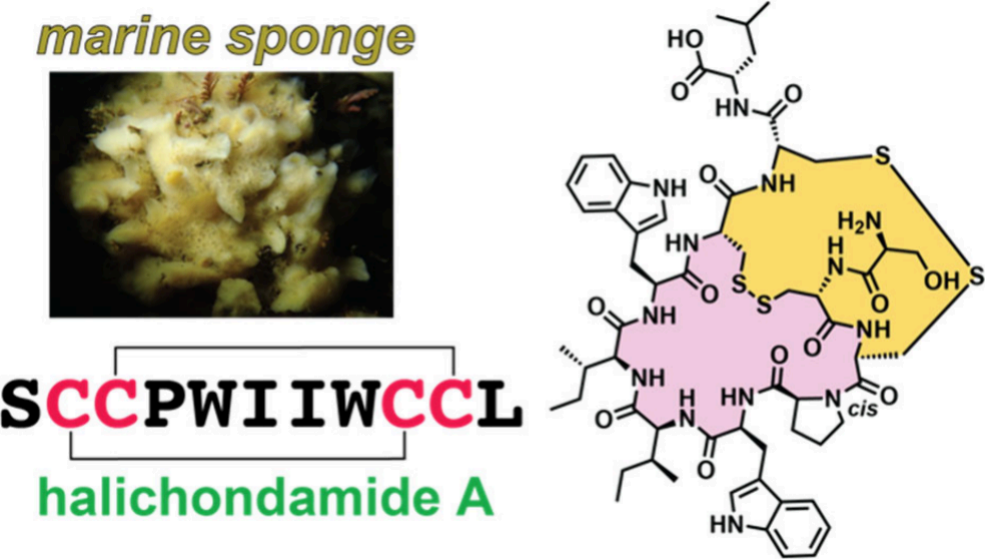

We describe the discovery and structure of an undecapeptide natural product from a marine sponge, termed halichondamide A, that is morphed into a fused bicyclic ring topology via two disulfide bonds. Molecular dynamics simulations allow us to posit that the installation of one disulfide bond biases the intermediate peptide conformation and predisposes the formation of the second disulfide bond. The natural product was found to be mildly cytotoxic against liver and breast cancer cell lines.

Peptidic natural products find widespread utility in the clinic, with the antibiotic vancomycin, immunosuppressant cyclosporine, anticancer actinomycin, and analgesic ziconotide being representative examples.^1^ In all of the above-mentioned examples, a conserved structural feature is macrocyclization in which the conformational flexibility of the peptide is constrained by covalent linkages involving the peptide main chain and/or the amino acid side chains. Macrocyclization confers rigidity, proteolytic stability, and facilitates transport through biological membranes.^2^

In the marine environment, filter-feeding sponges are prolific sources of natural products.^3^ Sponges are holobionts in which the eukaryotic host is associated with commensal and symbiotic microbiomes. All three components of the sponge holobiont are sources of macrocyclized peptidic natural products.^4^ Illustratively, the sponge host excises proline-rich macrocyclic peptides (PRMPs) from ribosomally synthesized precursor peptides while members of the commensal microbiome employ nonribosomal peptide synthetases (NRPSs) to produce macrocyclic peptidic natural products.^5−7^ The sponge symbiotic microbiome is also a site of macrocyclic peptide biosynthesis.^8^ Emergent techniques in mass spectrometry are driving the discovery of peptidic natural products, while (meta)genome sequencing technologies are connecting these molecules to their biosynthetic gene clusters.^9,10^ Herein, we describe the discovery of the peptidic natural product halichondamide A (**1**) from a marine sponge, evaluation of its bioactivity, and elaboration of a possible biosynthetic route (Figure 1).

**Figure 1:**
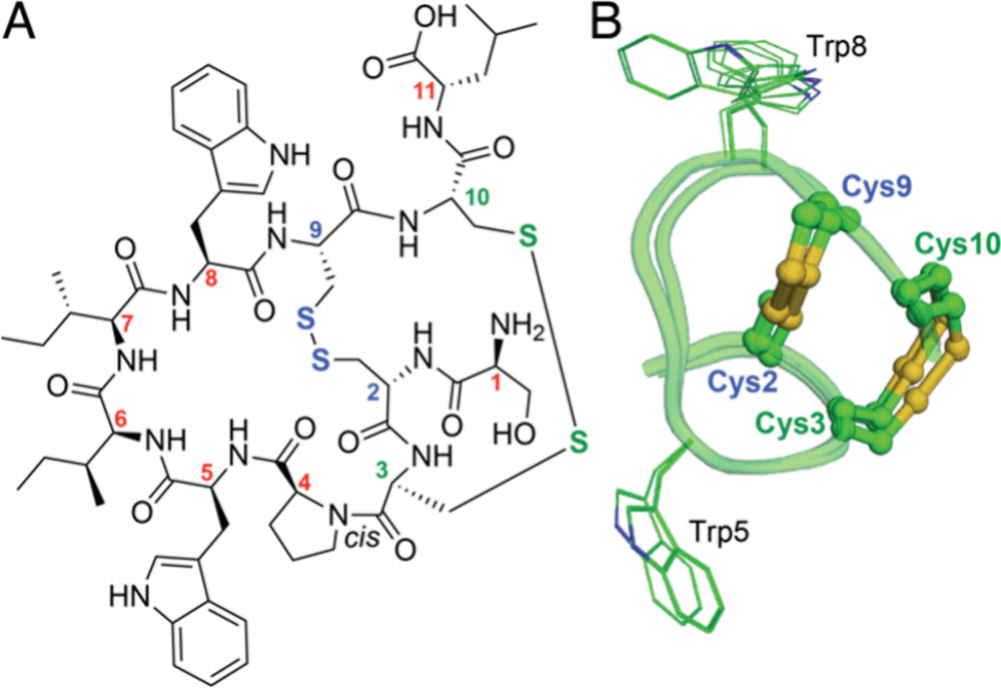
(A) Structure of **1** with Cα atoms numbered for clarity. (B) Overlaid 10 least energy structures of **1** derived from solution NMR. The Cys side chains are shown in stick-ball representation. The Trp side chains are shown as lines.

Screening the organic extract of the sponge *Halichondria bowerbanki* revealed the presence of several peptidic molecules (Figure S1 of the Supporting Information, SI). *H. bowerbanki* is an encrusting marine sponge in the Pacific Coast of North America where it is likely an introduced species.^11^ Fractionation of the organic extract allowed for the isolation of **1** as a white powder. Molecular formula for **1** indicated 26 degrees of unsaturation (Figure S2). The 1D ^1^H NMR spectrum revealed amide proton signals (δ_H_ 6.33–8.73 ppm) and amino acid Cα proton signals (δ_H_ 3.66–4.72 ppm) indicating it to be peptidic (Figure S3, Table S1). From the 1D ^13^C{^1^H} NMR spectra, the observation of amide carboxylic carbon signals (δ_C_ 167.6–175.3 ppm) and amino acid Cα carbon signals (δ_C_ 50.0–61.0 ppm) further supported the peptidic nature of **1** (Figure S4). On the basis of 2D ^1^H–^13^C HMBC correlations between the main chain amide protons or Cα protons and the neighboring carboxylic carbon atoms, dipeptide Ser^1^-Cys^2^ and nonapeptide Cys^3^-Pro^4^-Trp^5^-Ile^6^-Ile^7^-Trp^8^-Cys^9^-Cy^10^-Leu^11^ fragments were identified which satisfied 24 of the 26 degrees of unsaturation (Figures S5–S8). However, the lack of clear HMBC correlations between these two fragments hindered determination of the entire undecapeptide sequence.

Examination of the 2D ^1^H–^1^H ROESY spectrum revealed a through-space correlation between Cβ-proton of the Cys^3^ and the Cα-proton of the Cys^10^, indicating the presence of a disulfide bridge (Figure 2A). Consequently, the side chain thiols of Cys^2^ and Cys^9^ were also implicated to be involved in a second disulfide bond formation, as was deduced by the addition of 4H to the molecular mass upon reduction of **1** by tris(2-carboxyethyl)phosphine (TCEP) (Figure 2B). Treatment with the sulfhydryl alkylating reagent iodoacetamide did not result in derivatives for **1**; however, the TCEP-reduced product resulted in the addition of four acetamide units (Figures S9–S11). Examination of MS^2^ spectra of reduced-**1** with four acetamide additions indicated that the dipeptide and nonapeptide fragments were assembled as the Ser^1^-Cys^2^-Cys^3^-Pro^4^-Trp^5^-Ile^6^-Ile^7^-Trp^8^-Cys^9^-Cys^10^-Leu^11^ undecapeptide with disulfide bonds between the Cys^2^/Cys^9^ and Cys^3^/Cys^10^ side chain thiols (Figure S12). Identification of amino acid residues was further confirmed by 2D ^1^H–^13^C HMBC and ^1^H–^1^H COSY correlations (Figure 2C, Table S1). Furthermore, comparison of chromatography retention time and MS^1^ and MS^2^ spectra of reduced-**1** with the synthetic peptide standard corroborated the deduced peptide sequence (Figures S13–S15). Marfey’s analysis for reduced-**1** established all amino acids to be proteinogenic l-amino acids (S16–S20). The configuration of Pro residue was assigned *cis* based on the empirical rules that correlate with ^13^C{^1^H} NMR chemical shifts (δC_γ_ < 23.3 ppm) and [Δδ(C_β_–C_γ_) > 8.0 ppm].^12^

**Figure 2:**
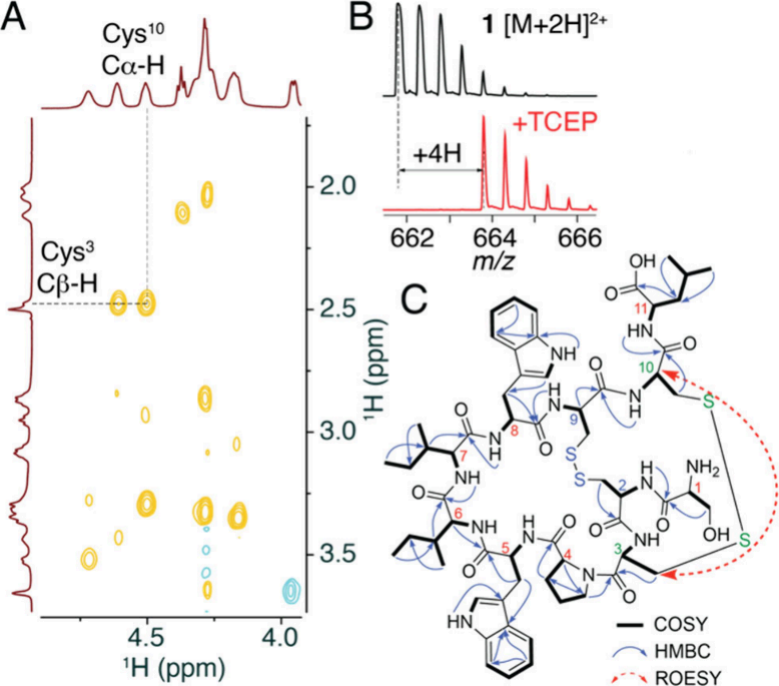
(A) Abbreviated ^1^H–^1^H ROESY spectra for **1** denoting the key correlation between Cys^3^ Cβ–H and Cys^10^ Cα–H. Positive (cross-peaks) and negative (diagonal) ROE signals are colored orange and blue, respectively. (B) Mass spectra demonstrating increase in mass corresponding to 4H upon reduction of **1** by TCEP. (C) Deduced chemical structure of **1** with ^1^H–^1^H COSY and ^1^H–^13^C HMBC correlations highlighted. The ROESY correlation highlighted in panel A is denoted by dashed red arrow.

Next, we determined the 3D solution structure of **1** by NMR spectroscopy (Table S2). The two disulfide bridges hold the peptide backbone of the fused bicyclic core of **1** in a rigid conformation (Figure 1B). Notably, unlike the disulfide-knotted peptides barrettides and asteropsins, Cys residues proximal to both the N- and C-termini of **1** are involved in disulfide bond formation which rigidifies the entire peptide backbone (Table S3).^13−17^ Thus, the peptide backbone rigidity of **1** more closely mimics that of asteropine A and the neopetrosiamides.^18−20^ The solution structure of **1** possesses no secondary structural elements.

All above-mentioned disulfide knotted peptides bear only proteinogenic amino acids consistent with sponge-derived ribosomal peptide precursors proposed for barretides.^17^ Thus, the biosynthetic origin of disulfide knotted peptides likely resembles that of PRMPs in which post-translational modification of sponge-derived ribosomal peptide precursors furnishes the natural product.^7^ While the emergence of the sponge eukaryotic host as a natural product biosynthetic factory is only a recent phenomenon,^7,21^ the presence of disulfide knotted peptides is spread across Eukarya with defensins, spider venoms, and conus snail venoms being prominent examples.^22−26^ The disulfide bonds in all of these natural products rigidify the peptide structure and lend it proteolytic stability. Alternatively, disulfide bonds in NRPS-derived peptidic natural products, such as romidepsin, serve as a redox-tunable metal chelating motif.^27^

Cysteine-rich peptide oxidation furnishes a mixture of isomers.^20^ However, only a single topological isomer is detected in the sponge extract. Hence, to construct **1**, it is plausible that macrocyclization is not unguided, and that enzymes chaperone the thiol/disulfide exchange which underlies disulfide bond formation.^28^ In a parsimonious biosynthetic scheme, the formation of the first disulfide bond must proceed with high fidelity to furnish a conformationally restricted intermediate, which can then bias the unguided self-assembly of the remaining disulfide bonds. Indeed, such a route has been chemically realized wherein chemically stapled peptides are restricted in their intramolecular disulfide bond formation outcomes.^29^ To query whether such a scenario could be operative in the biosynthesis of **1**, we computationally explored the conformations of two different partially reduced derivatives of **1** wherein only a singular disulfide bond was present between the Cys^2^/Cys^9^ residues or between Cys^3^/Cys^10^ residues.

Two independent molecular dynamics (MD) simulation trajectories over 1000 ns revealed lower root-mean-square deviations (RMSDs) for backbone atoms of the fully oxidized **1** and the hypothetical Cys^2^/Cys^9^-reduced derivative of **1** as compared to the Cys^3^/Cys^10^-reduced **1** (Figure 3). We also monitored the distances between the Cys^2^-Sγ and Cys^9^-Sγ atoms, and the Cys^3^-Sγ and Cys^10^-Sγ atoms. For wild-type **1**, these distances remain near constant at ∼2 Å (Figure 3A, right). When the Cys^3^/Cys^10^ disulfide bond was reduced, the ensuing backbone RMSDs along the MD simulations were much greater as compared to when the Cys^2^/Cys^9^ disulfide bond was reduced, implying a greater conformational flexibility for the Cys^3^/Cys^10^-reduced derivative (Figure 3B, 3C, middle panels). Furthermore, preinstallation of the Cys^3^/Cys^10^ disulfide bond on average leads to a much shorter interatomic distance between the Cys^2^-Sγ and Cys^9^-Sγ atoms (Figure 3B, right), as compared to the interatomic distance between the Cys^3^-Sγ and Cys^10^-Sγ atoms when the Cys^2^/Cys^9^ disulfide bond was preinstalled (Figure 3C, right). These observations were corroborated by a principal component analyses of peptide conformations wherein the Cys^2^/Cys^9^-reduced peptide adopts conformations much closer to that of **1**, while the conformations of the Cys^3^/Cys^10^-reduced peptide are more divergent (Figure S21). Furthermore, the φ/ψ dihedral angles for the different conformations sampled by the Cys^2^/Cys^9^ partially reduced peptide mimic that for **1**, while the dihedral angles for the Cys^3^/Cys^10^ partially reduced peptide are different (Figure S22–S24). Taken together, these data allow us to posit that the Cys^3^/Cys^10^ disulfide bond is likely installed first with the aid of an enzyme/chaperone. Subsequently, the then biased conformation of the intermediate facilitates the subsequent installation of the Cys^2^/Cys^9^ disulfide bond as a biosynthetic cascade.^30^ This, then, represents the substrate-assisted catalysis model for maintaining topological fidelity during the biosynthesis of cysteine knotted peptides.^31^ The hypothesis presented here remains to be experimentally tested.

**Figure 3:**
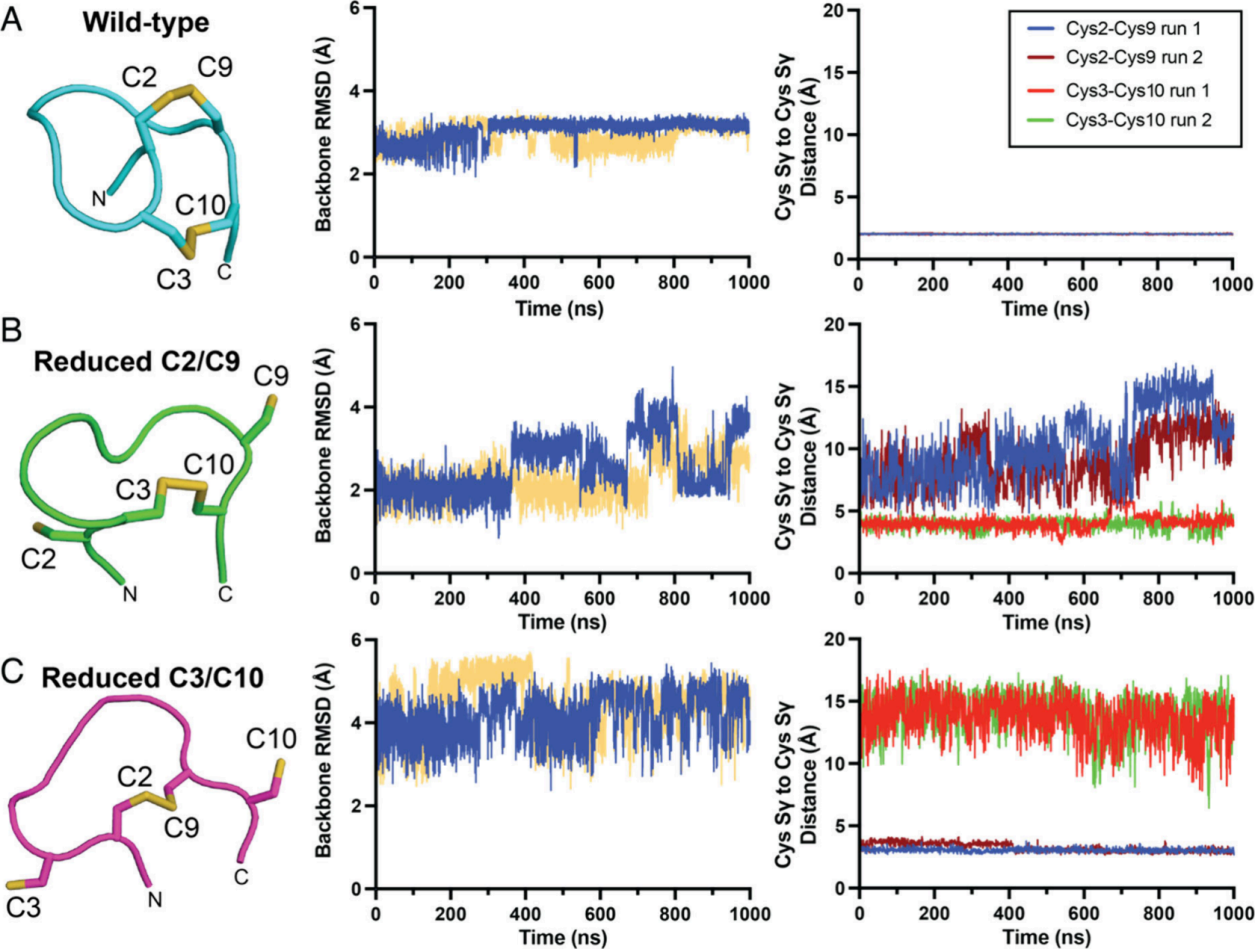
(A, left) Structure of **1**. (A, middle) Overall backbone RMSD over 1000 ns of simulation time. (A, right) Distance between the Cys^2^/Cys^9^ and Cys^3^/Cys^10^ Sγ atoms across simulation time. (B, left) Structure of partially reduced **1** wherein the Cys^2^/Cys^9^ disulfide bond has been reduced. (B, middle) The overall backbone RMSD for the Cys^2^/Cys^9^-reduced derivative of **1**. (B, right) Distance between the Cys^2^/Cys^9^ and Cys^3^/Cys^10^ Sγ atoms across simulation time for the Cys^2^/Cys^9^-reduced derivative of **1**. (C, left) Structure of partially reduced **1** wherein the Cys^3^/Cys^10^ disulfide bond has been reduced. (C, middle) The overall backbone RMSD for the Cys^3^/Cys^10^-reduced derivative of **1**. (C, right) Distance between the Cys^2^/Cys^9^ and Cys^3^/Cys^10^ Sγ atoms across simulation time for the Cys^3^/Cys^10^-reduced derivative of **1**. For RMSD calculations, backbone atoms are defined as N, Cα and CO of the peptide backbone.

Molecule **1** demonstrated mild cytotoxic activity against both hepatic (HepG2 and HuH-7) and breast (MDA-MB-231) cancer cell lines (Figure 4). While the aforementioned liver cancer cell lines exhibit low metastatic potential,^32^ MDA-MB-231 cells possess invasive behavior and are known to be prone to metastasis.^33^

**Figure 4:**
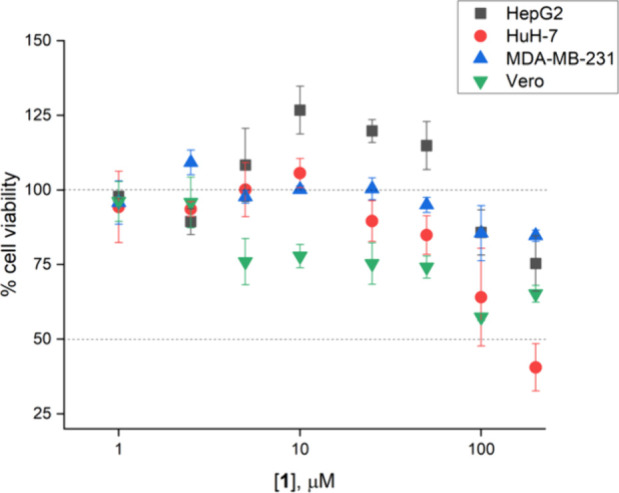
Quantification of cell death/viability upon treatment with **1**. Response data were obtained after 72 h treatment of liver (HepG2, HuH-7), and breast (MDA-MB-231) cancer cell lines with **1**. Vero, a normal kidney cell line, was included for comparison. Each data point represents mean and errors bars depict standard deviation from three independent measurements.

Cysteine knotted peptides likely follow the biosynthetic paradigm of PRMPs wherein a ribosomal precursor peptide produced by the eukaryotic sponge is morphed into a peptidic natural product. It is plausible that these peptidic natural products play roles in core sponge physiology, and/or in defensive interactions with their ecological neighbors, as have been established for other marine peptidic natural products.^34^ This would be in line with the entomological application of cysteine knotted peptides as venoms in the terrestrial and marine realms. It is thus tantalizing to propose that the peptidic cysteine knot as a structural motif has survived evolutionary diversification across Eukarya due to its primal relevance in organismal physiology and chemical ecology.

## Experimental Section

The detailed experimental section is available in the SI. Structural assignments were performed based on additional information from gHSQC, gCOSY, and gHMBC correlations.

## Data Availability

The data underlying this study are available in the published article and its SI. The mass spectrometry fragmentation data for **1** have been deposited to the Global Natural Product Social Molecular Networking (GNPS) library with the annotation ID CCMSLIB00012451692. Coordinates for the three-dimensional structure of **1** have been deposited to the Protein Data Bank (PDB) with accession ID 9BHN and the NMR data have been deposited to the Natural Product Magnetic Resonance Database (NP-MRD) and the Biological Magnetic Resonance Bank (BMRB) with accession numbers NP0332808 and 31170, respectively.

## Supporting Information Available

Experimental details, Figures S1–S24, and Tables S1–S3 (PDF)
