# Supplementary material for: Discovery and Folding Dynamics of a Fused Bicyclic Cysteine Knot Undecapeptide from the Marine Sponge Halichondria bowerbanki
Source: J Org Chem. 2024 Aug 27;89(17):12748–52. doi: 10.1021/acs.joc.4c01104 (PMC11382151; doi:10.1021/acs.joc.4c01104)
Supplement: Supplementary file 1 — jo4c01104_si_001.pdf [file jo4c01104_si_001.pdf]

Supporting Information for:

Discovery and folding dynamics of a fused bicyclic cysteine knot undecapeptide from the marine sponge *Halichondria bowerbanki*

Weimao Zhong,[1] Jeremiah O. Olugbami,[1,2] Prashanth Rathakrishnan,[1] Ipsita Mohanty,[1] Samuel G. Moore,[3] Neha Garg,[1] Adegboyega K. Oyelere,[1] Thomas Turner,[4] Andrew C. McShan,[1,*] and Vinayak Agarwal[1,5,*]

[1]School of Chemistry and Biochemistry, Georgia Institute of Technology, Atlanta, GA 30332, USA

[2]Department of Biochemistry, University of Ibadan, Ibadan, Oyo 200005, Nigeria

[3]Petit Institute for Bioengineering and Bioscience, Georgia Institute of Technology, Atlanta, GA 30332, USA

[4]Ecology, Evolution, and Marine Biology Department, University of California Santa Barbara, Santa Barbara, CA 93106, USA

[5]School of Biological Sciences, Georgia Institute of Technology, Atlanta, GA 30332, USA

*Correspondence: andrew.mcshan@chemistry.gatech.edu; vagarwal@gatech.edu

This Supporting Information document contains:

Supplementary Materials and Methods

Supplementary Tables S1–S3

Supplementary Figures S1–S24

Supplementary References

Supplementary Materials and Methods

General Experimental Procedures

All chemicals, solvents, and media components were purchased from Sigma-Aldrich, Fisher Scientific, and VWR, and used without further purification. Optical rotation, circular dichroism, and UV spectra were measured on a JASCO J-815 spectropolarimeter (JASCO). One-dimensional (1D) and two-dimensional (2D) NMR spectra were acquired on Bruker AVIII-HD 800 and AVIII-HD 700 MHz NMR. The ^{1}H and $^{13}C\{^{1}H\}$ NMR chemical shifts were referenced to the solvent peaks for DMSO-d_6 at δ_H 2.50 ppm and δ_C 39.52 ppm. The chemical shift (δ) values are given in parts per million (ppm), and the coupling constants (*J* values) are in Hz. High resolution electrospray ionization mass data were recorded on a 1290 Infinity II ultra-performance liquid chromatography (UPLC, Agilent Technologies) coupled to a Bruker ImpactII ultra-high-resolution Q-ToF mass spectrometer equipped with an electron spray ionization (ESI) source (Bruker Daltonics). A Kinetex C18 reverse phase UHPLC column (50 x 2.1 mm, 1.7 μm) was used for chromatographic separation. Data were acquired in positive ionization mode with *m/z* 50–2000 Da. Low resolution electrospray ionization mass data were recorded on a 1260 Infinity high performance liquid chromatography (HPLC, Agilent Technologies) coupled to a Bruker amaZon mass spectrometer equipped with an electron spray ionization (ESI) source (Bruker Daltonics). A Poroshell 120 EC-C18 reverse phase HPLC column (100 x 4.6 mm, 5.0 μm) was used for chromatographic separation. Data were acquired in negative ionization mode with *m/z* 50–2000 Da. Open column chromatography (CC) was performed over a C18 SPE cartridge (Phenomenex). Semi-preparative HPLC was performed on an Agilent 1260 Infinity II HPLC system equipped with a VWD detector, using a Luna C18 reverse phase column (250*10 mm, 5 μm). All solvents used in CC and HPLC were analytical (VWR) and HPLC grades (Fisher and Sigma-Aldrich). Synthetic peptide SCCPWIIWCCL was obtained from GenScript.

Sponge Material

The marine sponge *Halichondria bowerbanki* was collected from kelp forest habitat at a depth of 5 m at Coal Oil Point, Santa Barbara, California, USA. The species was identified by digesting subsamples of sponge tissue in bleach and examining spicules, and identification was confirmed with phylogenetic analysis of DNA sequences. Sequences are vouchered in GenBank under the numbers PP729067 and PP734164.

Extraction and Purification

The sponge (0.2 kg, dry weight) was minced and exhaustively extracted with a mixture of methanol and CH_2Cl_2 (v/v 1:1). Extraction was evaporated to dryness in vacuum. The crude extract (1.0 g) was subjected to a C18 SPE open column using stepwise gradient elution with MeOH/Water (v/v, 5:95, 50:50, 100:0, each 200 mL) to yield 3 fractions (1–3). Fraction 3 (210 mg) was further fractionated using semipreparative HPLC [C_{18} column, 250×10 mm, 5 μm, 50% MeCN/H_2O (+0.1% v/v TFA) at 2 mL/min flowrate] to yield **1** (5.0 mg).

Halichondamide A (**1**): White solid; [α]25 D= -104.5 (*c* 2.0, MeOH); UV (MeOH) λ_{max} (log ε) 230 (4.2), 281 (4.0); ECD (MeOH) λ_{max} ($\Delta\varepsilon$) 205 (-39.9), 212 (11.8), 215 (9.1), 224 (-15.1); ^{1}H and ^{13}C NMR data, Table S1; HRMS (ESI-TOF) *m/z*: $[M + H]^+$ Calcd for $C_{60}H_{84}N_{13}O_{13}S_4$ 1322.5189; Found 1322.5175.

Reduction and Iodoacetamide Derivatization

To a solution of 0.5 mg of compound **1** in 1.0 mL water in a 4 mL vial was added 100 µL of 18 mM tris(2-carboxyethyl)phosphine (TCEP). The mixture was stirred at room temperature and reduction of **1** was monitored by LC-MS until the disappearance of the starting material. Half of the reaction solution was transferred to a separate 4 mL vial and dried by lyophilization for further Marfey's analysis (see below). To the rest of the reaction mixture was added 20 µL of 1.0 M iodoacetamide and 50 µL of triethylamine (0.726 g/mL). This mixture was stirred at room temperature and monitored by LC-MS until the disappearance of the starting material. Compound **1**, without reduction, was treated with iodoacetamide and trimethylamine in the same condition as a control.

Marfey's Analysis

The TCEP-reduced **1** (250 µg) was hydrolyzed using 6 N HCl (1.0 mL) at 110 ˚C in a 4 mL vial. 100 µL reaction volume was taken at different reaction times (2 h, 3 h, 4 h, 5 h, 6 h, 7 h, 8 h, 9 h, and 24 h). The reaction aliquots were dried *in vacuo* and resuspended in dH_2O (100 µL), to which was added 10% w/v 1-fluoro-2-4-dinitrophenyl-5-L-alanine amide (L-FDAA) in acetone (33 µL) and 1 N $NaHCO_3$ in dH_2O (50 µL). These reactions were incubated at 40 ˚C for 1 h. The derivatization reactions were quenched by adding 1 N HCl (50 µL), dried *in vacuo*. Residues were dissolved in MeOH (0.2 mL) prior to analysis by LC-MS. Amino acid standards were prepared and analyzed in an identical manner.

Solution NMR

Halichondamide A (**1**, SCCPWIIWCCL) purified from the sponge as described above was dissolved at 20 mg/mL in 20% DMSO-d_6 + 80% $CDCl_3$ at a volume of 0.25 mL and transferred to a 3 mm NMR tube, and at 10 mg/mL in 100% DMSO-d_6 at a volume of 0.5 mL and put into a 5 mm NMR tube, respectively. NMR spectra were acquired on a Bruker AVIII HD 800 MHz and AVIII HD 700 MHz spectrometer both equipped with a triple-resonance inverse (TCI) cryoprobe at 298.15 K. Standard Bruker 2D ^{1}H–^{1}H COSY (pulse sequence cosygpppqf), 2D ^{1}H–^{1}H TOCSY (pulse sequence dipsi2esgpph), 2D ^{1}H–^{1}H ROESY (pulse sequence roesyphpp.2), 2D ^{1}H–^{13}C carbon heteronuclear single quantum coherence (HSQC) (pulse sequence hsqcetgpsisp2.2), 2D ^{1}H–^{15}N HSQC (pulse sequence hsqcetgpsi2), and 2D ^{1}H–^{13}C carbon heteronuclear multiple bond coherence (HMBC) (pulse sequence hmbcgplpndqf) were acquired with gradients. The TOCSY mixing time was 80 msec, and the ROESY mixing time was 300 msec. The relaxation delay was 1.5 sec.

MestReNova (version 6.0) was used to perform NMR chemical shift assignment, and CYANA (version 2.1) was used for the structural calculation.[1] $^1H–^1H$ distance restraints, automatically assigned by CYANA and confirmed manually, were used as restraints in the structural calculation. A total of 200 structures were calculated, after seven iterations, using the software's simulated annealing protocol. Ten structures with the lowest energy were chosen to produce the final ensemble. Final structures were checked in MolProbity. Images of structures were generated with PyMOL (version 2.5.5).

Cell Culture

Cells were cultured in their respective media as follows: Hep-G2 cells in MEM; HuH-7 cells in low-glucose DMEM; and MDA-MB-231 and Vero cells in high-glucose DMEM. Media were supplemented with 10% fetal bovine serum, 1% Pen/Strep, and 1% L-glutamine (excluding media for MDA-MB-231 and Vero cells).

MTT Assay

Following the method of Yadava *et al.*[2] with minor modifications, cells, at a density of 4.5 x 10^3 cells/well and a volume of 100 µL, were seeded in 96-well tissue culture plates and allowed to attach for 24 h. Thereafter, the culture medium was removed, and the cells were treated for 72 h with 100 µL of varying concentrations (1 – 200 µM) of molecule **1**, dissolved in dimethyl sulfoxide but diluted with the respective culture media. Subsequently, 10 µL of MTT reagent (5 mg/mL) was added to the culture medium and the plates were incubated for 3 h, after which the MTT reagent/medium was carefully aspirated. Finally, formazan crystals formed were dissolved with molecular grade DMSO (100 µL/well) and absorbance was measured at 570 nm using multimode plate reader (Tecan Infinite M200 Pro, Männedorf, Switzerland).

Molecular Dynamics Simulations

All atom molecular dynamics (MD) simulations were performed explicit solvent using the solution NMR structure of halichondamide A (**1**, model 1 from PDB ID 9BHN). Simulations were performed on the wild-type halichondamide A (all cysteines oxidized), reduced C2/C9 halichondamide A (reduced disulfide bond between Cys2 and Cys9, while Cys3/Cys10 was oxidized), and reduced C3/C10 halichondamide A (reduced disulfide bond between Cys3 and Cys10, while Cys2/Cys9 was oxidized). For reduced structures, target disulfide bonds were broken in PyMOL using the *unbond* command. Inputs for MD were separately prepared in CHARMM-GUI Solution Builder Module in the Input Generator using the CHARMM36 force field and TPI3P water model.[3] The halichondamide A peptides were solvated in an aqueous environment using default parameters in CHARMM-GUI, and sodium and chloride ions were added to the aqueous system at concentrations that naturally occur in the human body (150 mM). Default CHARMM-GUI periodic boundary conditions were used, and the thermodynamic ensemble was nPT. Temperature was set constant at 298.15 K and maintained using the

Nosé–Hoover coupling method with a tau-t of 1 psec. An integration time step of 2 fsec was used with coordinates output every 10 psec. The LINCS algorithm was used to constrain H-bonds. An isotropic Parrinello–Rahman method with a tau-p of 5 psec and a compressibility of 4.5×10^{-5} bar^{-1} was used for pressure coupling. Short range interactions were treated with a Verlet cutoff scheme with 10 Å electrostatic and van der Waals cutoffs and long-range electrostatics were treated using the particle mesh Ewald method. MD simulations were performed in GROMACS v2021.5 with NVIDIA Tesla V100 GPUs on the Phoenix Cluster provided to us by the Partnership for an Advanced Computing Environment at the Georgia Institute of Technology.[4] Simulations were run in independent duplicate replicates for a total of 1000 nanoseconds each. After the simulations concluded, the simulation trajectories were visualized and analyzed through GROMACS and the Visual Molecular Dynamics (VMD) program v1.9.3.[5] Backbone RMSD for peptides were plotted in VMD. Ramachandran plots of each Cys residue throughout MD trajectories were performed in VMD. Other commands for MD simulation analysis performed GROAMCS are provided below.

<u>Distance Measurements between Sγ atoms of Cys residues</u>

center the trajectory

```
echo "1 0" | gmx trjconv -s step5_1_production.tpr -f step5_1_production.xtc -o centered.xtc -pbc mol -ur compact -center
```

creating index file

```
echo "1 q" | gmx make_ndx -f step5_1_production.tpr -o step5_1_production.ndx
```

note atom IDs for the cysteine residues (SG) and put each pair in a file called distance_selections

```
echo "atomnr 21 141" >> distance_selections
echo "atomnr 31 151" >> distance_selections
```

calculation of distances in nm

```
cat distance_selections | gmx distance -n step5_1_production.ndx -f centered.xtc -s step5_1_production.tpr -oall distances.xvg -tu ns -dt 0.5 -b 0 -e 1000 -len 1.4 -tol 1 -binw 0.01
```

Principal component analysis

covariance matrix calculation, with 4:backbone selected for least squares fit and covariance when prompted

```
gmx covar -s step5_1_production.tpr -f centered.xtc -o eigenvalues.xvg -v
eigenvectors.trr
```

#analysis of eigenvectors, selecting 4:backbone for least squares fit and the indices that correspond to the eigenvectors when prompted

```
gmx anaeig -s step5_1_production.tpr -f centered.xtc -v eigenvectors.trr
-first 1 -last 2 -proj projection.xvg
```

SUPPLEMENTARY TABLES

Table S1: ^{1}H (800 MHz) and ^{13}C{^{1}H} (201 MHz) NMR chemical shifts of **1** in 20% DMSO-d_6 + 80% $CDCl_3$ (*J* in Hz, *δ* in ppm)

Halichondamide A (**1**)					
Residue	No.	δ_H (*J*, Hz)[a]	δ_C, type[b]	HMBC	ROESY
Ser[1]	1		167.6, C		
	2	4.19, overlap	54.0, CH		
	3	4.28, overlap	60.4, CH_2	1, 2	
		3.95, d (8.1)			
	NH	nd			
	OH	nd			
Cys [2]	1		170.0, C		
	2	4.72, s	53.8, CH		
	3	3.52, d (12.6)	42.0, CH_2	1	
		3.30, overlap			
	NH	8.73, s		2, 3, Ser[1]-1	Ser[1]-H2
Cys[3]	1		170.5, C		
	2	4.61, s	50.0, CH	3	Pro[4]-H2
	3	2.86, overlap	38.7, CH_2	1	
		2.48, overlap			Cys[10]-H2
	NH	8.57, br s			
Pro[4]	1		168.6, C		
	2	4.29, overlap	61.0, CH	1, 3, 4, 5	
	3	2.04, overlap	29.5, CH_2	1, 2, 4, 5	
		1.55, overlap			
	4	1.31, m	20.3, CH_2	2	
		0.59, m			
	5	3.09, m	45.1, CH_2	3, 4, Cys[3]-1	
		2.00, overlap			
Trp[5]	1		171.8, C		
	2	4.17, overlap	55.4, CH	1, 3	
	3	3.35, overlap	26.6, CH_2	1, 2, 4, 5, 10	
		3.07, overlap			
	4		108.3, C		
	5	7.20, s	124.9, CH		
	6	7.27, d (7.9)	111.0, CH	8, 10	
	7	7.00, t (7.1)	121.0, CH	9, 11	
	8	6.92, t (7.5)	118.5, CH	6, 10	
	9	7.42, d (7.6)	117.6, CH	7, 11	
	10		125.9, C		
	11		136.3, C		
	NH	6.79, s			Pro[4]-H2
	Indole -NH	10.31 s		4, 10, 11	
Ile[6]	1		175.3, C		
	2	4.37, t (10.2)	56.0, CH	1, 3, 4, 6, Trp[5]-1	
	3	2.12, m	35.4, CH	1, 2, 5, 6	
	4	1.54, overlap	24.2, CH_2	2, 3, 5, 6	
		1.17, overlap			
	5	0.89, overlap	10.1, CH_3	3	
	6	0.70, d (7.4)	14.8, CH_3	2, 3, 4	
	NH	7.63, overlap			

Ile[7]	1		173.6, C		
	2	3.66, s	61.0, CH	1, 3, 4, 6	
	3	1.53, overlap	34.5, CH	1, 4, 6	
	4	1.08, m<br>0.96, m	25.1, CH_2	2, 3, 5, 6	
	5	0.67, t (7.1)	10.8, CH_3	3, 4	
	6	0.40, d (5.1)	14.2, CH_3	2, 3, 4	
	NH	8.69, s		1, 2, 3, Ile[6]-1	Ile[6]-H2
Trp[8]	1		172.9, C		
	2	4.29, overlap	54.7, CH	4	
	3	3.30, overlap<br>2.87, overlap	26.5, CH_2	1, 2, 4, 5	
	4		107.7, C		
	5	6.94, s	122.8, CH	3, 4, 10, 11	
	6	7.30, d (8.1)	111.3, CH	8, 10	
	7	7.06, t (7.7)	121.4, CH	9, 11	
	8	6.96, t (7.2)	118.7, CH	6, 10	
	9	7.39, d (7.6)	117.4, CH	7, 11	
	10		126.0, C		
	11		136.4, C		
	NH	6.33, s		Ile[7]-1	Ile[7]-H2
	Indole-NH	10.35, s		4, 5, 10, 11	
Cys[9]	1		170.3, C		
	2	4.33, overlap	54.9, CH	1	
	3	3.44, s<br>3.28, overlap	42.8, CH_2		
	NH	7.60, overlap		2, 3, Trp[8]-1	Cys[10]-NH
Cys[10]	1		169.4, C		
	2	4.51, s	50.2, CH	1, 3	
	3	3.29, overlap<br>2.95, t (13.1)	38.2, CH_2	1, 2	
	NH	7.88, s		3, Cys[9]-1	Leu[11]-NH
Leu[11]	1		173.7, C		
	2	4.28, overlap	50.5, CH	1, 3	
	3	1.65, overlap<br>1.58, overlap	39.8, CH_2	1, 2, 5, 6	
	4	1.67, overlap	24.1, CH	3	
	5	0.89, overlap	22.7, CH_3	3, 4, 6	
	6	0.81, d (5.9)	20.9, CH_3	3, 4, 5	
	NH	6.93, overlap		2, 3, Cys[10]-1	

[a]Recorded at 800 MHz. [b]Recorded at 201 MHz. [nd]Not detected.

Table S2: NMR and refinement statistics for halichondamide A (**1**)

	**Halichondamide A PDB ID 9BHN**
**NMR distance and dihedral constraints**	
Distance constraints	
Total NOE	42
Intra-residue	18
Inter-residue	0
Short range ($\|i - j\| = 1$)	30
Medium-range ($\|i - j\| < 4$)	6
Long-range ($\|i - j\| > 5$)	6
Intermolecular	0
Hydrogen bonds	0
Total dihedral angle restraints	
ϕ	10
ψ	10
**Structure statistics**	
Violations (mean and s.d.)	4.9
Distance constraints (Å)	0.86
Dihedral angle constraints (º)	0
Max. dihedral angle violation (º)	0
Max. distance constraint violation (Å)	0
Deviations from idealized geometry	
Bond lengths (Å)	0
Bond angles (º)	0
Impropers (º)	0
Average pairwise r.m.s. deviation** (Å)	
Heavy	1.26
Backbone	0.71

Table S3: Sequences and linkages of natural product peptides with disulfide bonds.

Compounds	Structures	References
halichondramide A (**1**)	SCCPWIIWCCL	This study
barrettide A	DVSPCFCVEDETSGAKTCVPDNCDASRGTNP	6
barrettide B	DVSPCFCVEDETSGAKTCLPDNCDASRGTNP	6
barrettide C	NVVPCFCVEDETSGAKTCIPDNCDASRGTNP	7
barrettide D	DVSPCFCVEDERSGAKNCLPDNCDASRGTNP	7
barrettide E	NVGPCFCVEDETSGAKMCIPDNCDASRGTNP	7
barrettide F	NVVPCFCVEDETSGAKTCVPDNCDASRGTNP	7
barrettide G	DVSPCFCVEDETSGAKTCIPDNCDASRGTNP	7
asteropsin A	XGCAFEGESCNVQFYPCCPGLGLTCIPGNPDGTCYYL	8
asteropsin B	XGCAFEGESCNVEFYPCCPGLGLTCIPGNPDGTCYYL	9
asteropsin C	XDCPGEGEQCDVEFNPCCPPLTCIPGDPYGICYII	9
asteropsin D	XGCAGPGEECIVGFYDCCPGYRCYPGDPGGICY	9
asteropsin E	CPGEGEQCDVEFNPCCPPLTCIPGDPYGICYII	10
asteropsin F	CPGEGEECDVEFNPCCPPLTCIPGDPYGICYII	11
asteropsin G	XWCAEEGESCEVYPCCDGLICYPTFPEPICGV	11
asteropine A	YCGLFGDLCTLDGTLACCIALELECIPLNDFVGICL	12
neopetrosiamide A/B	FFCPFGCALVDCGPNRPCRDTGFM*SCDC	13-14
gombamide A	XCPPFCZ	15

M*: methionine sulfoxide

X: pyroglutamic acid

Z: para-hydroxystyrylamide

SUPPLEMENTARY FIGURES

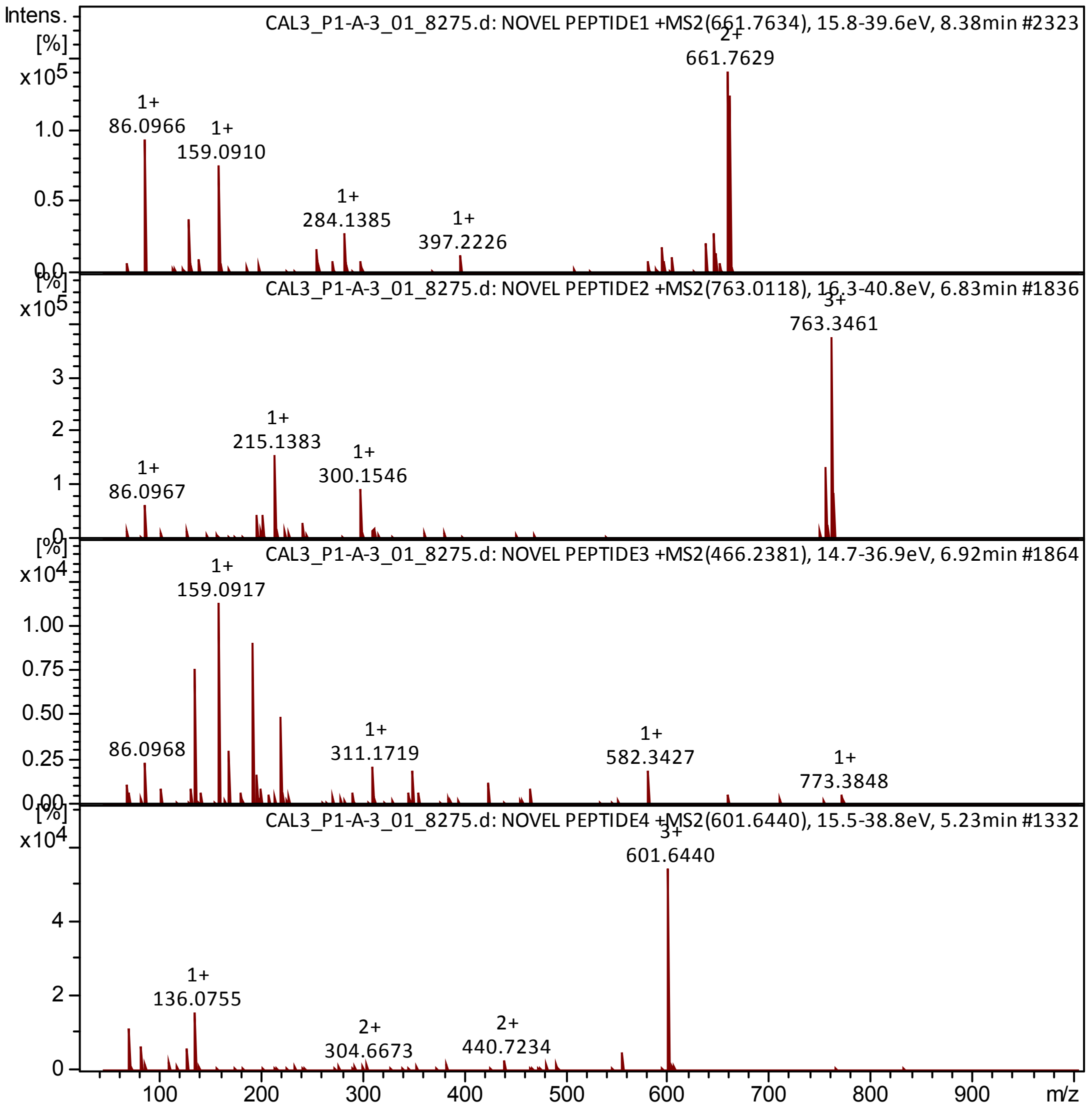

Figure S1: MS^2 fragmentation spectra for four different molecular species detected in the organic extract of the marine sponge *Halichondria bowerbanki*. The spectrum on the top corresponds to **1**. Fragment ions at *m/z* 86, *m/z* 159, *m/z* 136, among others, correspond to amino acid immonium ions. In all these spectra, neutral losses corresponding to proteogenic amino acids can be annotated.

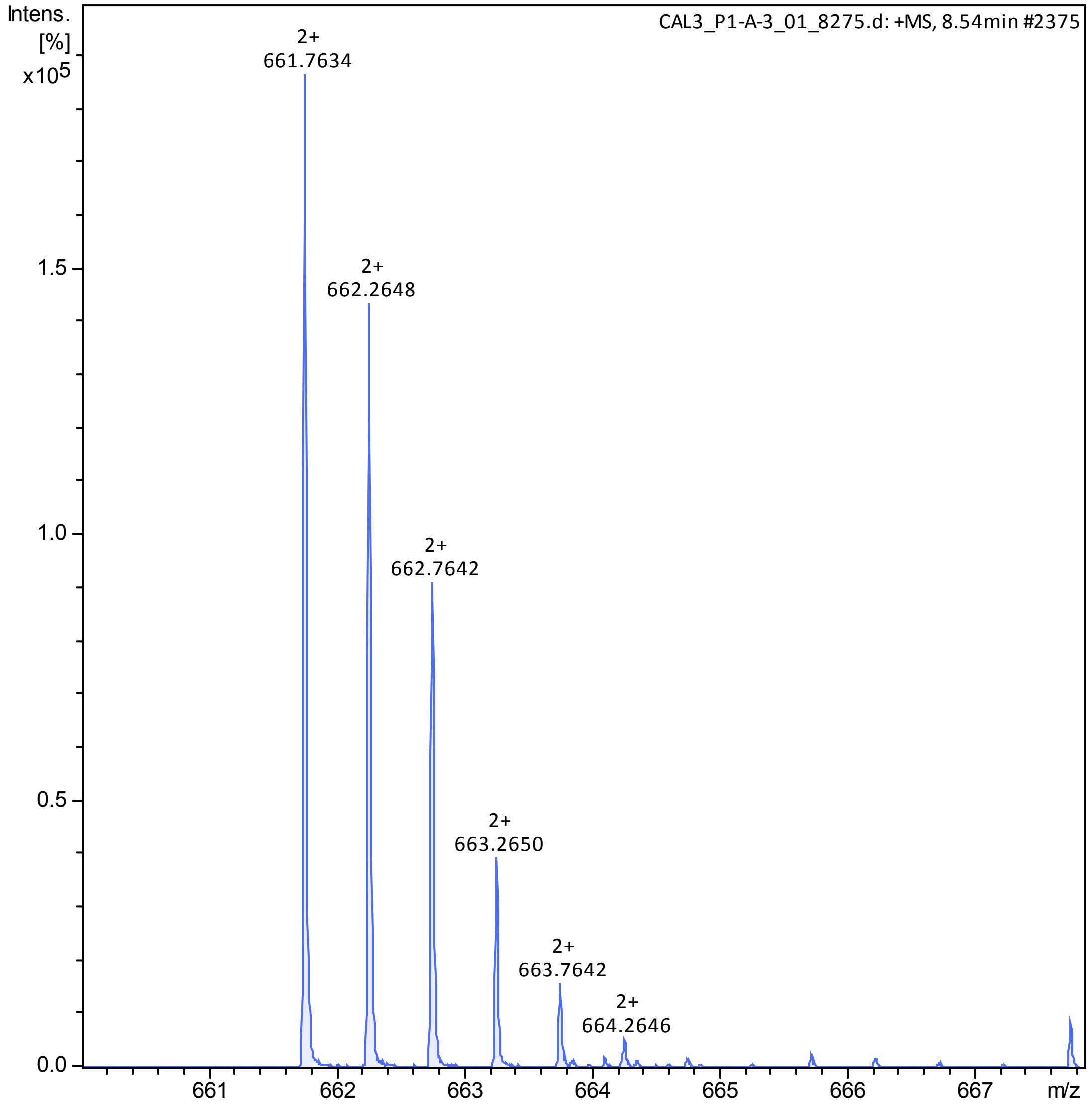

Figure S2: MS^1 spectra for the $[M+2H]^{2+}$ ion corresponding to **1**.

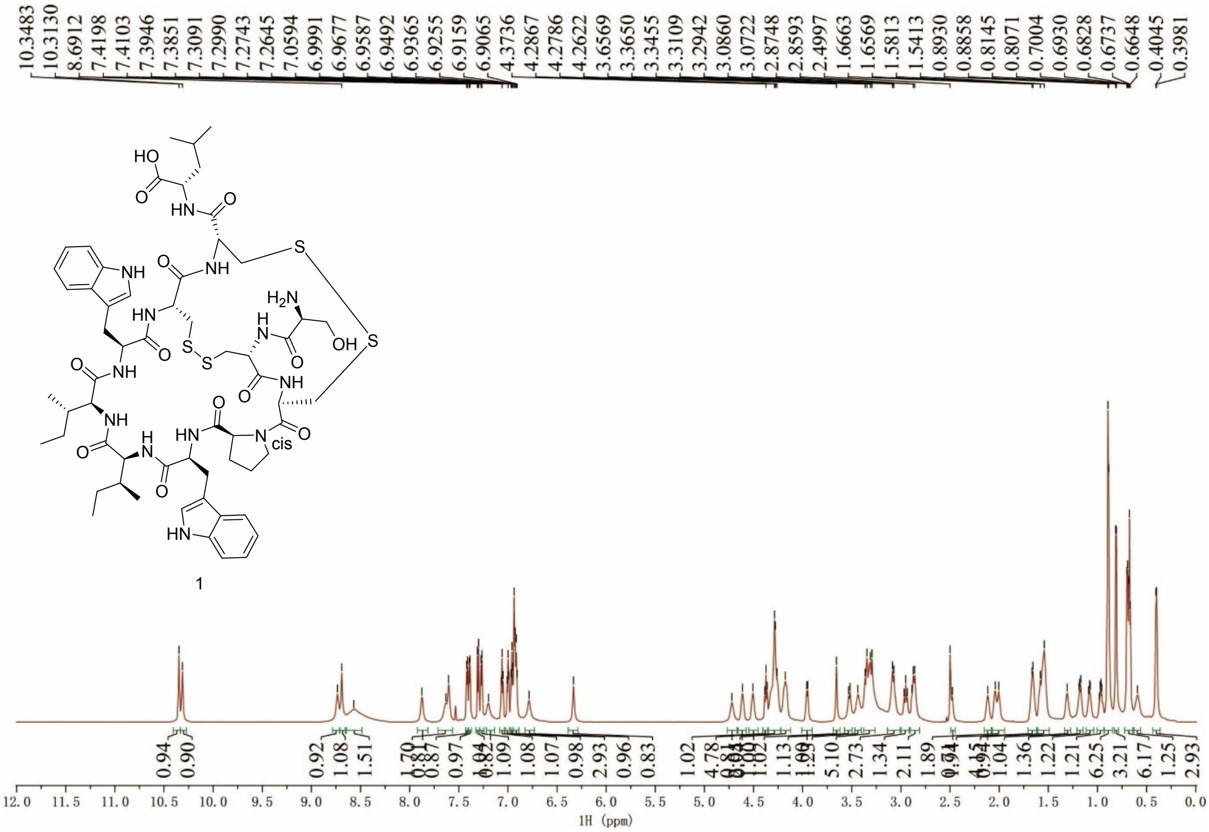

Figure S3. The ^{1}H NMR spectrum of **1** (800 MHz, 20% DMSO-d_6 + 80% $CDCl_3$).

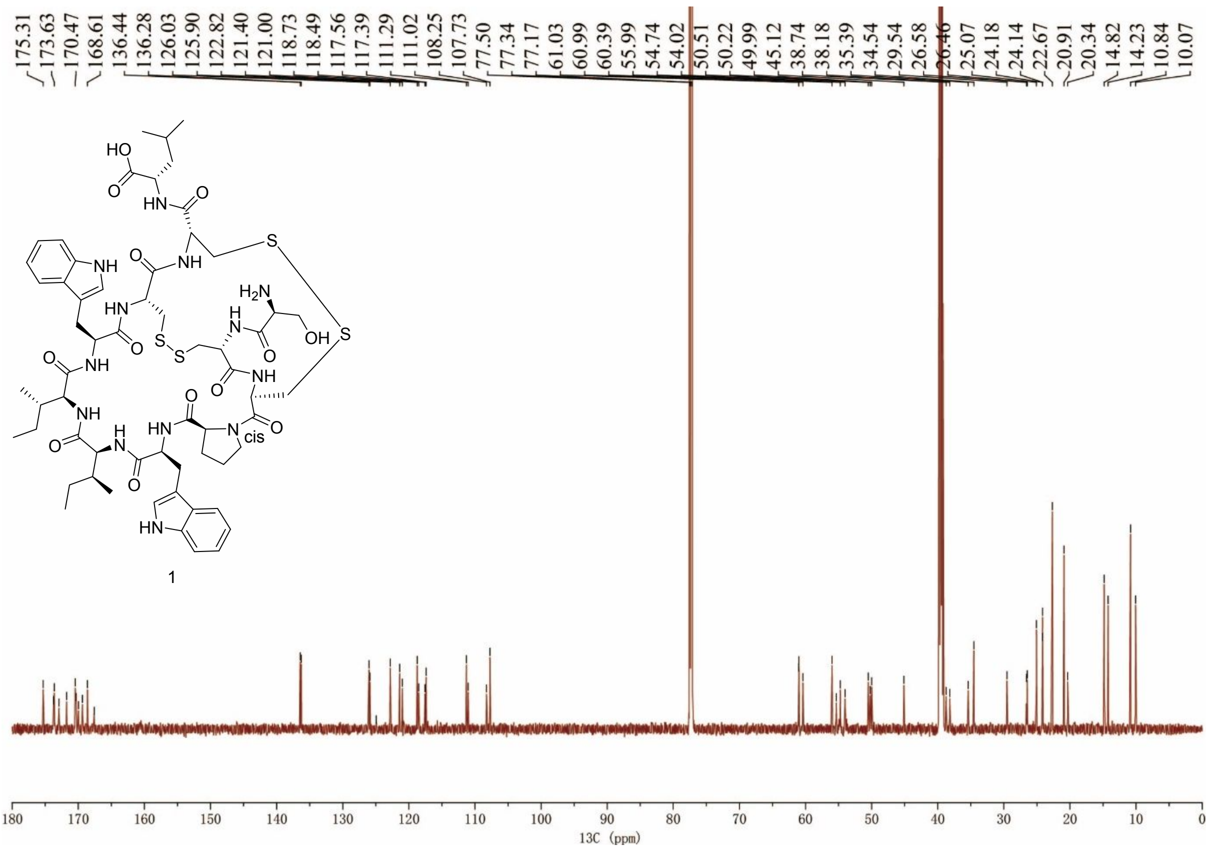

Figure S4. The $^{13}C\{^{1}H\}$ NMR spectrum of **1** (201 MHz, 20% DMSO-d_6 + 80% $CDCl_3$).

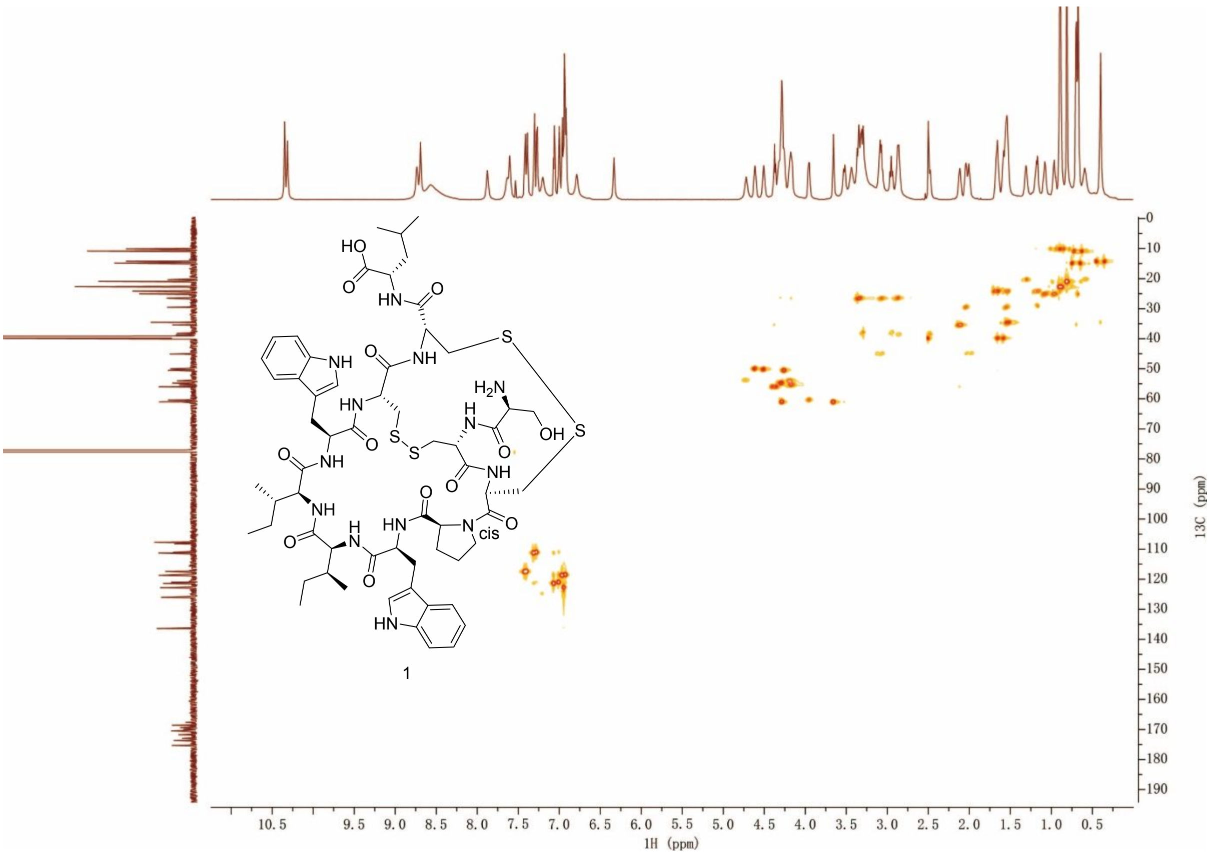

Figure S5. 2D ^{1}H-^{13}C HSQC spectrum of **1** (800 MHz, 20% DMSO-d_6 + 80% $CDCl_3$).

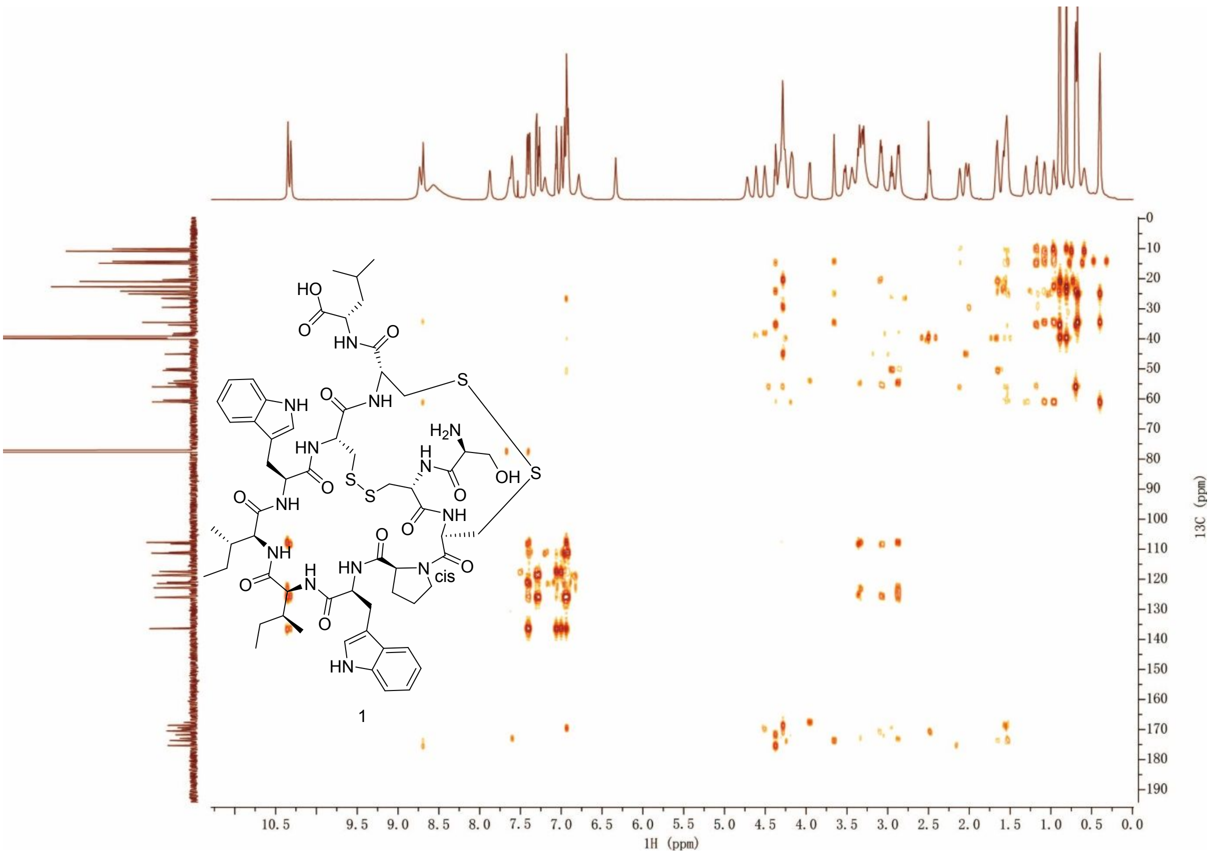

Figure S6. 2D ^{1}H-^{13}C HMBC spectrum of **1** (800 MHz, 20% DMSO-d_6 + 80% $CDCl_3$).

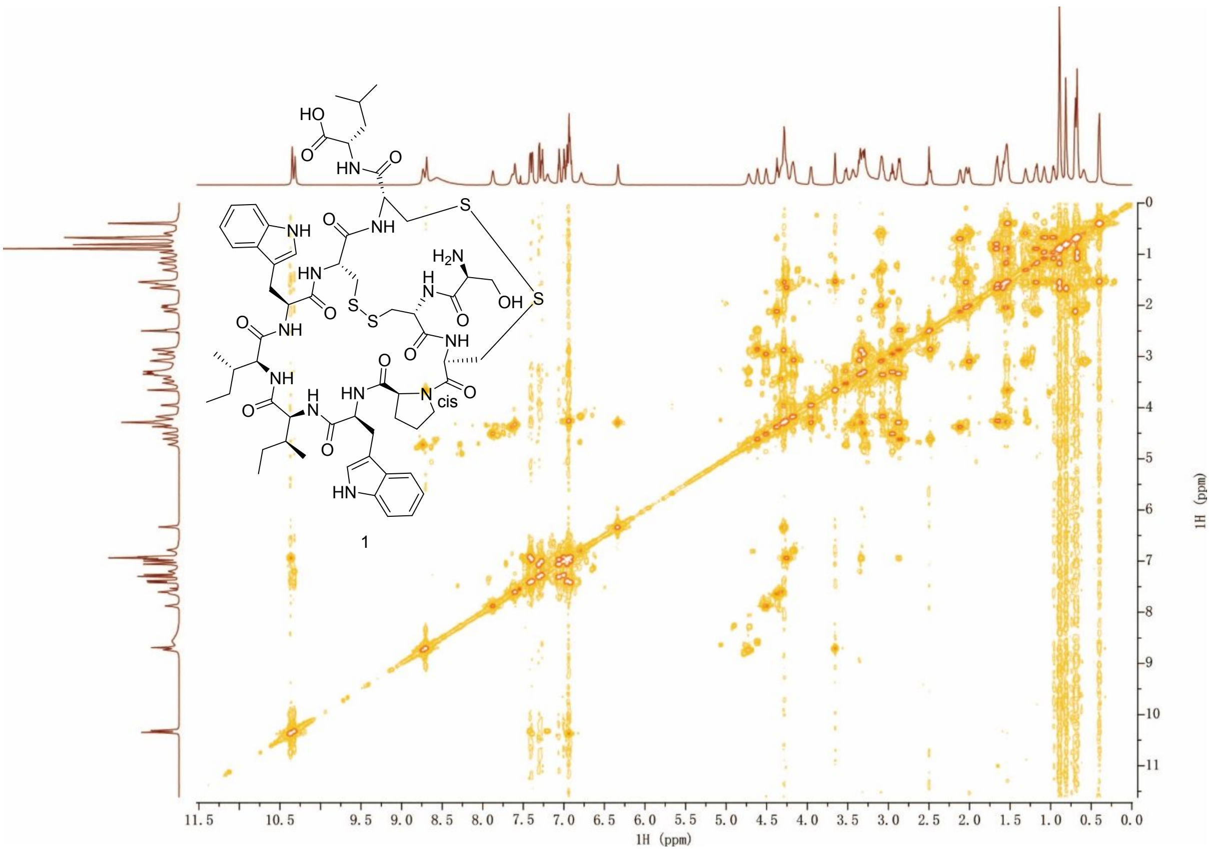

Figure S7. 2D ^{1}H-^{1}H COSY spectrum of **1** (800 MHz, 20% DMSO-d_6 + 80% $CDCl_3$).

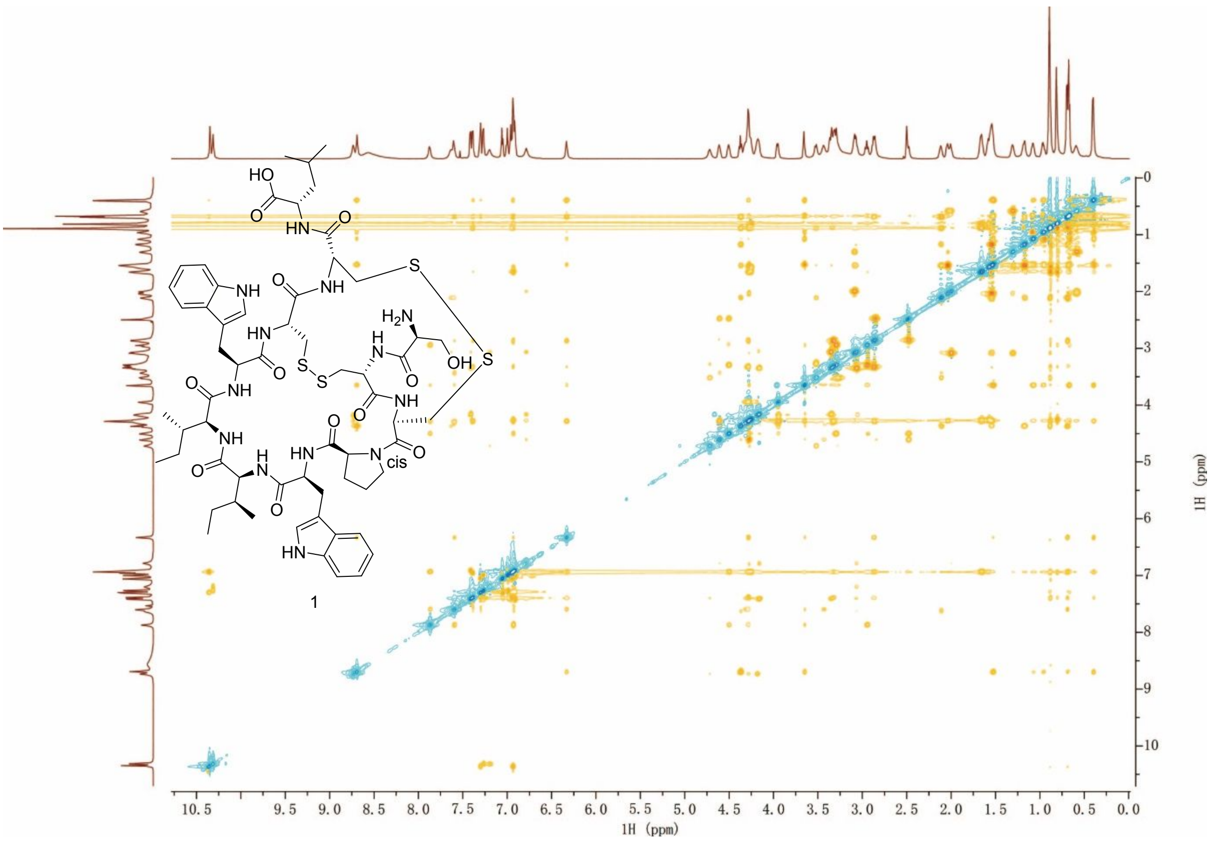

Figure S8. 2D ^{1}H-^{1}H ROESY spectrum of **1** (800 MHz, 20% DMSO-d_6 + 80% $CDCl_3$). Positive ROE cross-peaks are colored orange; ROE diagonals (self-ROEs) are colored blue.

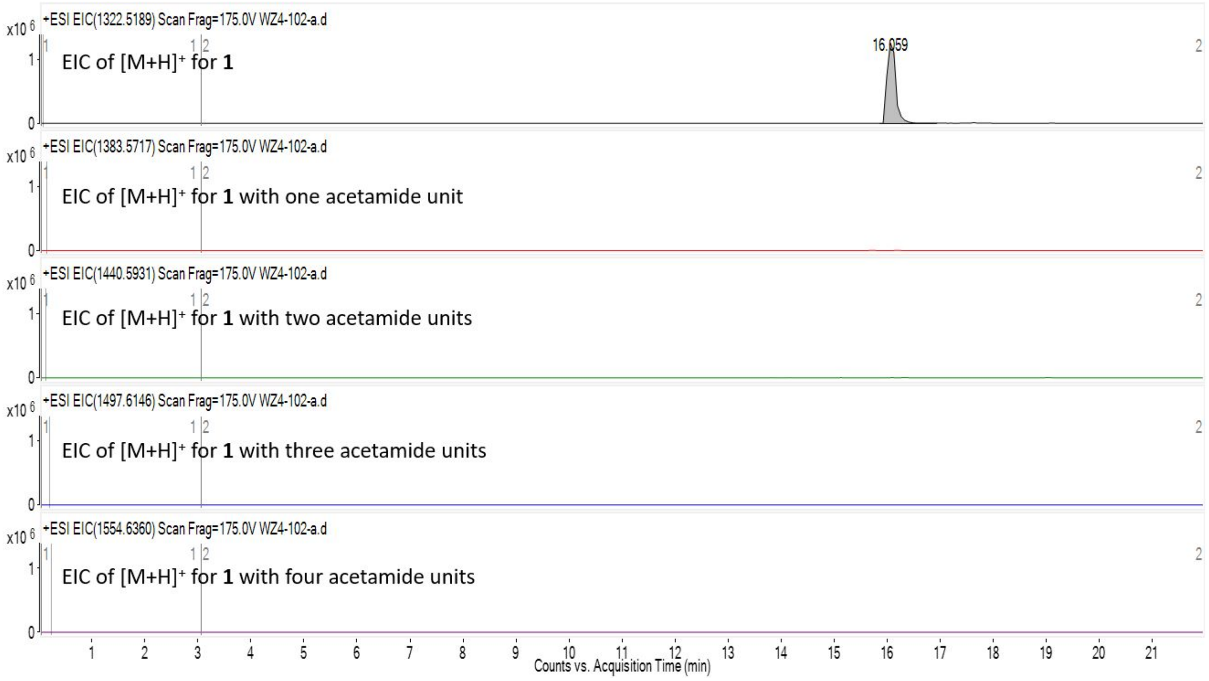

Figure S9. Extracted ion chromatograms (EICs) for the $[M+H]^+$ ions corresponding to compound **1** (top) and possible derivatives of **1** with different number of acetamide unit additions. Compound **1** was treated with iodoacetamide and the addition of different number of acetamide units was queried.

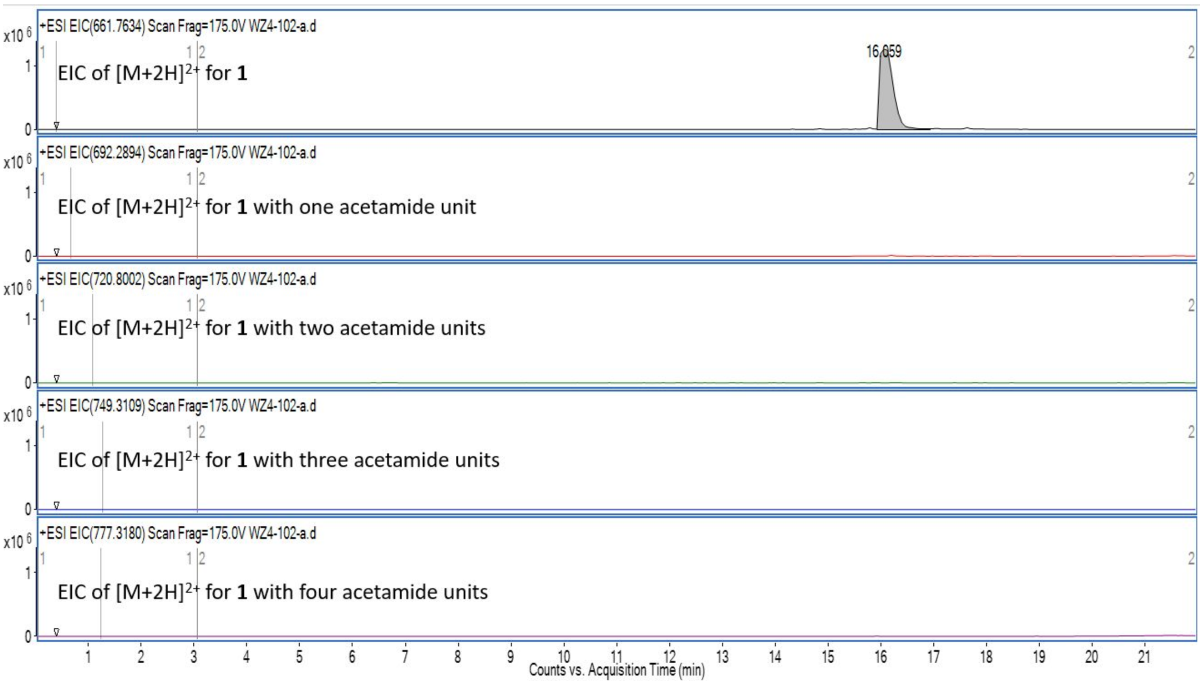

Figure S10. EICs for the $[M+2H]^{2+}$ ions corresponding to compound **1** (top) and possible derivatives of **1** with different number of acetamide unit additions. Compound **1** was treated with iodoacetamide and the addition of different number of acetamide units was queried.

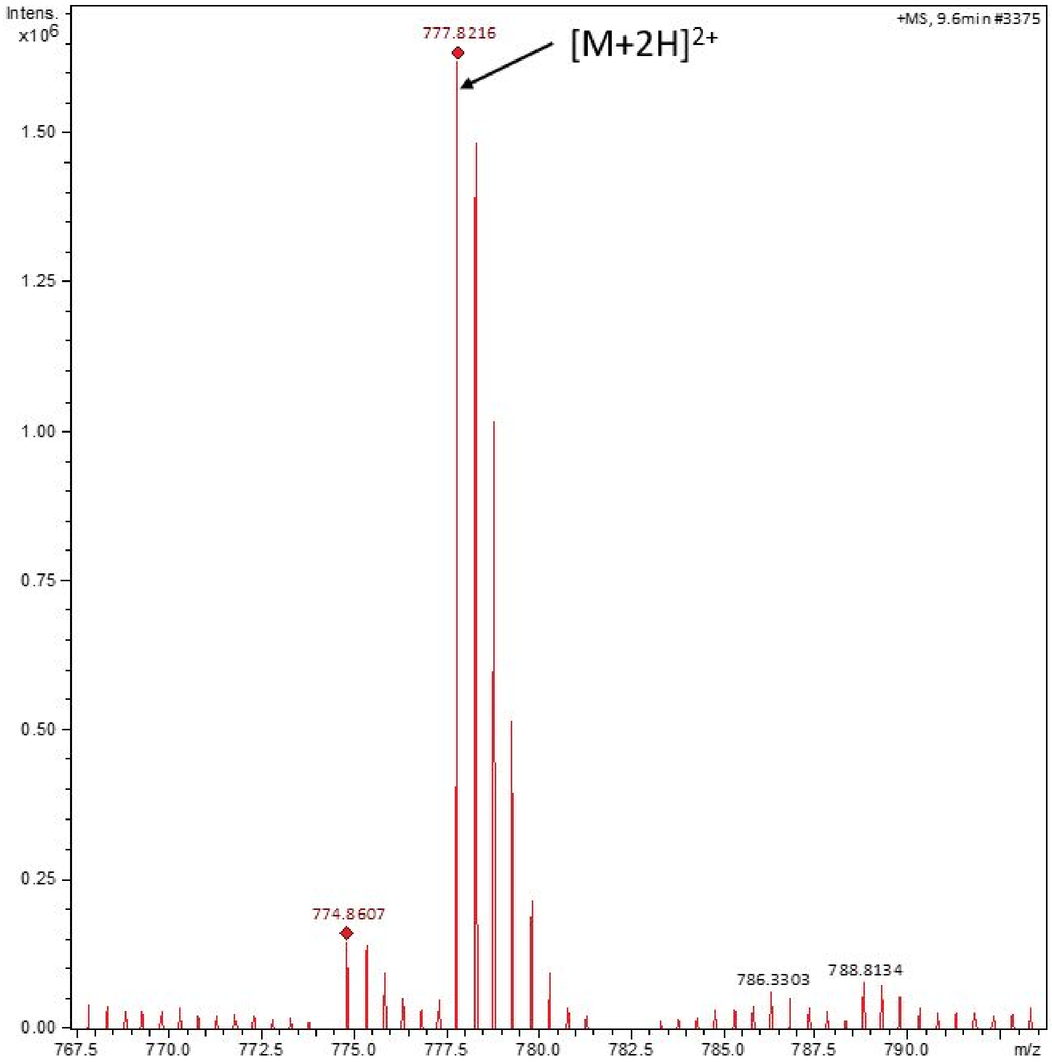

Figure S11. MS[1] spectra for the $[M+2H]^{2+}$ ion corresponding to TCEP-reduced **1** with four acetamide units.

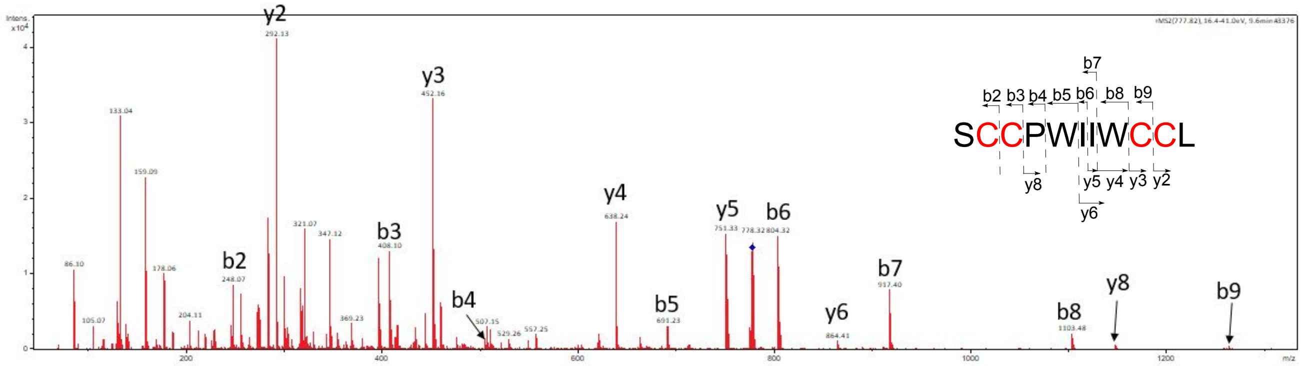

Figure S12. Annotation of MS^2 spectra ($[M+2H]^{2+}$ parent ion) of TCEP-reduced **1** with the four Cys side chain thiols labeled with acetamide additions.

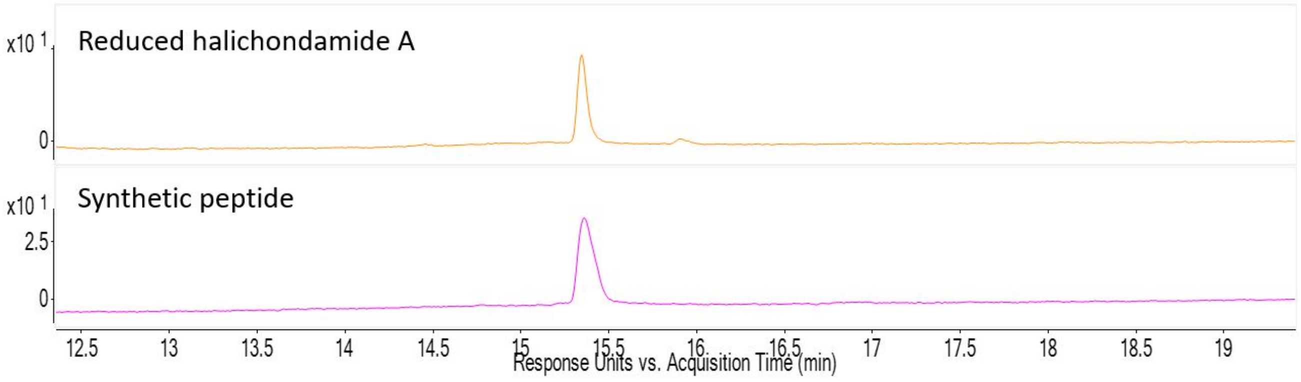

Figure S13. Comparison of retention time of TCEP-reduced halichondamide A (**1**) and synthetic SCCPWIIWCCL peptide. UV absorbance chromatograms were recorded at 280 nm wavelength.

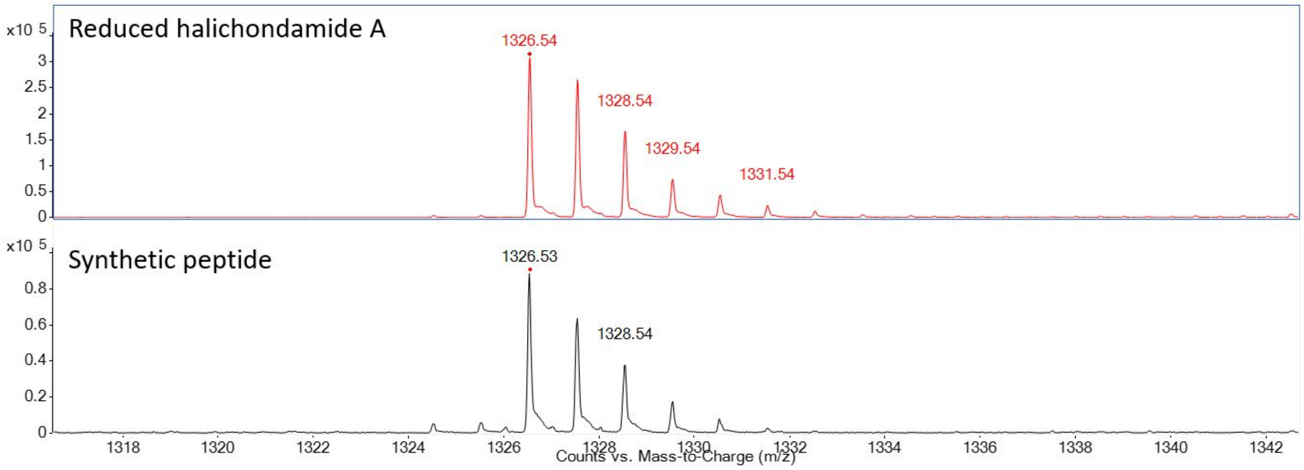

Figure S14. Comparison of the isotopic MS[1] signatures for the $[M+1H]^{1+}$ ions for TCEP-reduced halichondamide A (**1**) and synthetic SCCPWIIWCCL peptide.

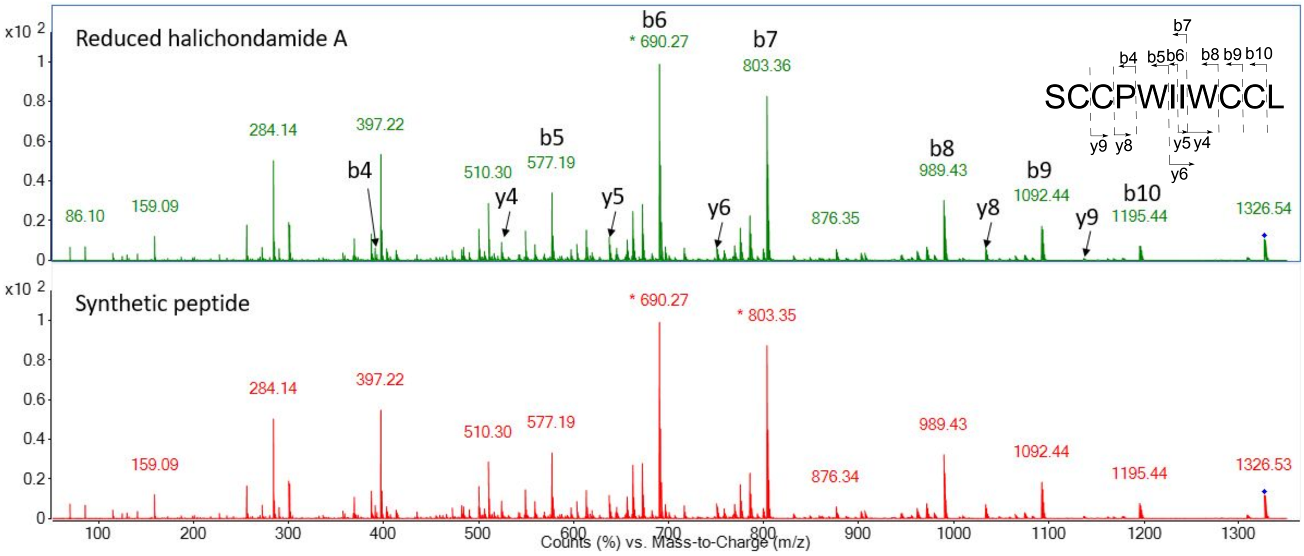

Figure S15. Comparison of MS^2 spectra ($[M+1H]^{1+}$ parent ions) for TCEP-reduced halichondamide A (**1**) and synthetic SCCPWIIWCCL peptide. The b- and the y-ions are annotated.

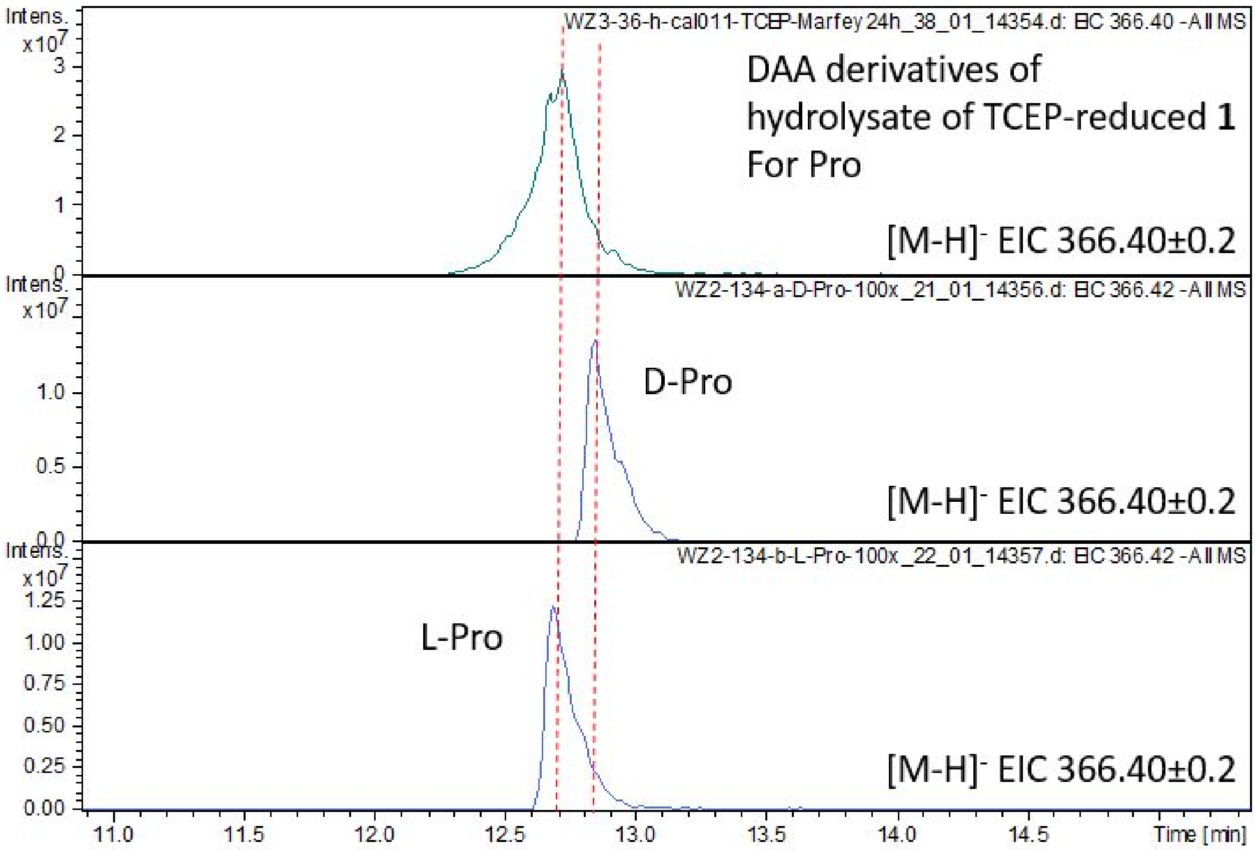

Figure S16. Marfey's analysis to determine the absolute configuration of the Pro residue in TCEP-reduced **1**. Extracted ion chromatograms (EICs) demonstrating the retention time of the DAA-derivatized Pro residue resulting from the acid hydrolysis of TCEP-reduced **1** (top), retention time of DAA-derivatized standard of D-Pro (middle), and the retention time of the similarly derivatized standard of L-Pro (bottom). Separation was achieved using the Agilent Poroshell EC-C18 (100×4.6 mm, 2.7 μm) column. Mass spectrometry data were acquired in the negative ionization mode.

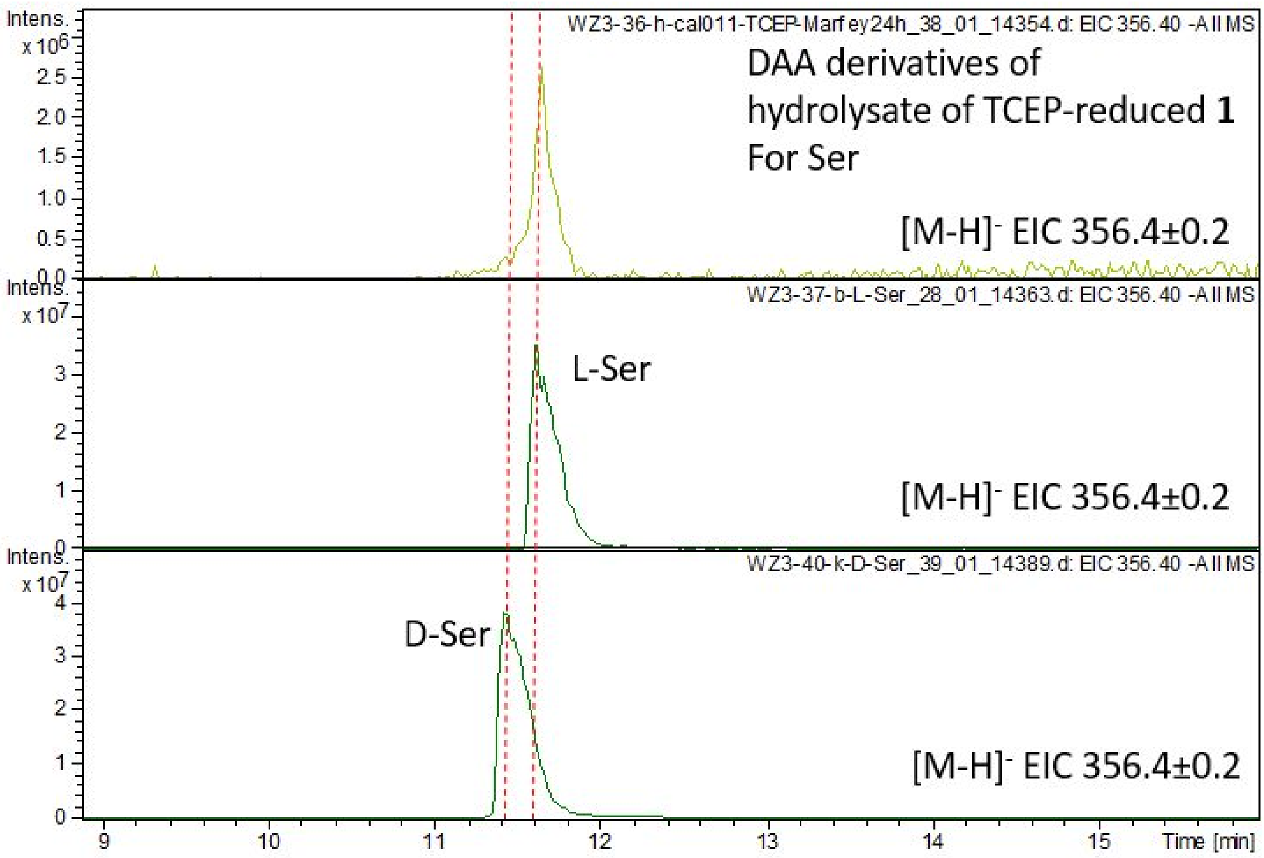

Figure S17. Marfey's analysis to determine the absolute configuration of the Ser residue in TCEP-reduced **1**. EICs demonstrating the retention time of the DAA-derivatized Ser residue resulting from the acid hydrolysis of TCEP-reduced **1** (top), retention time of DAA-derivatized standard of L-Ser (middle), and the retention time of the similarly derivatized standard of D-Ser (bottom). Separation was achieved using the Agilent Poroshell EC-C18 (100×4.6 mm, 2.7 µm) column. Mass spectrometry data were acquired in the negative ionization mode.

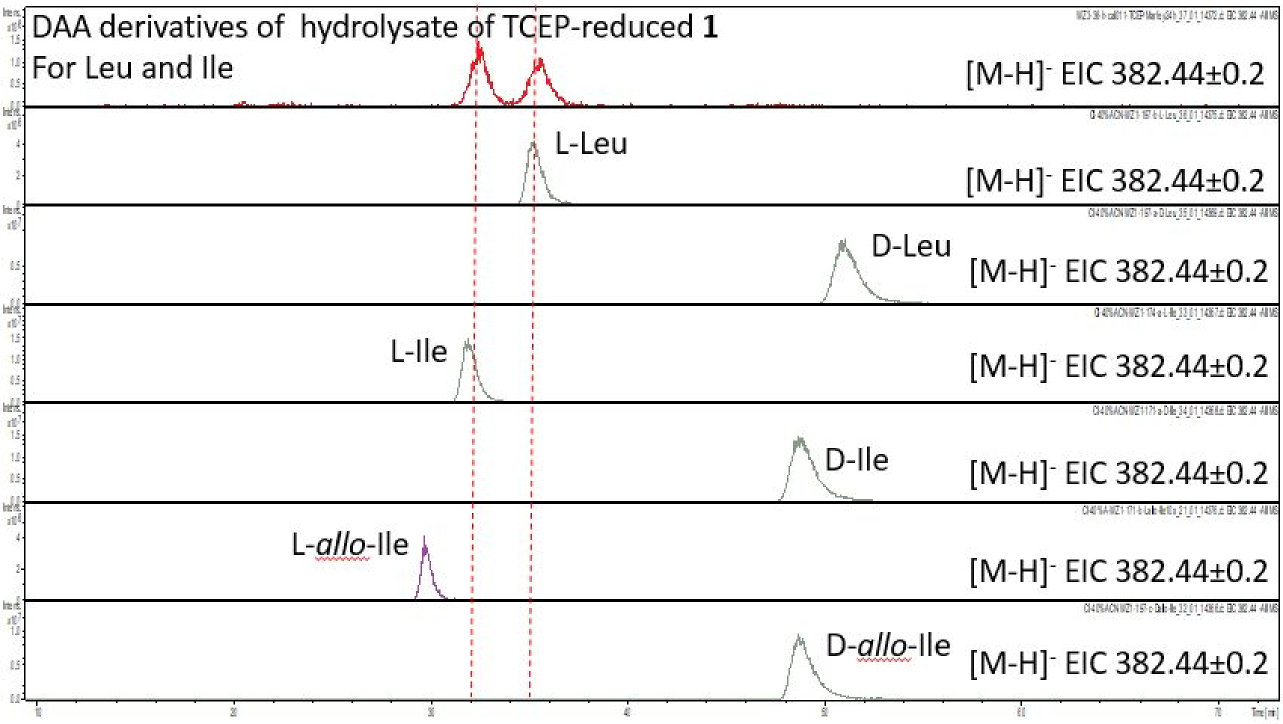

Figure S18. Marfey's analysis to determine the absolute configurations of the Leu and Ile residues in TCEP-reduced **1**. From top to bottom– EICs demonstrating retention time of DAA-derivatized Leu and Ile residues obtained by acid hydrolysis of TCEP-reduced **1**, DAA-derivatized standard for L-Leu, DAA-derivatized standard for D-Leu, DAA-derivatized standard for L-Ile, DAA-derivatized standard for D-Ile, DAA-derivatized standard for L-*allo*-Ile, and DAA-derivatized standard for D-*allo*-Ile. By retention time matching, the Leu and Ile residues in TCEP-reduced **1** were determined to be L-Leu and L-Ile, respectively. Chromatographic separation was achieved using the Cosmosil Cholester (250×4.6 mm, 5 μm) column. Mass spectrometry data were acquired in the negative ionization mode.

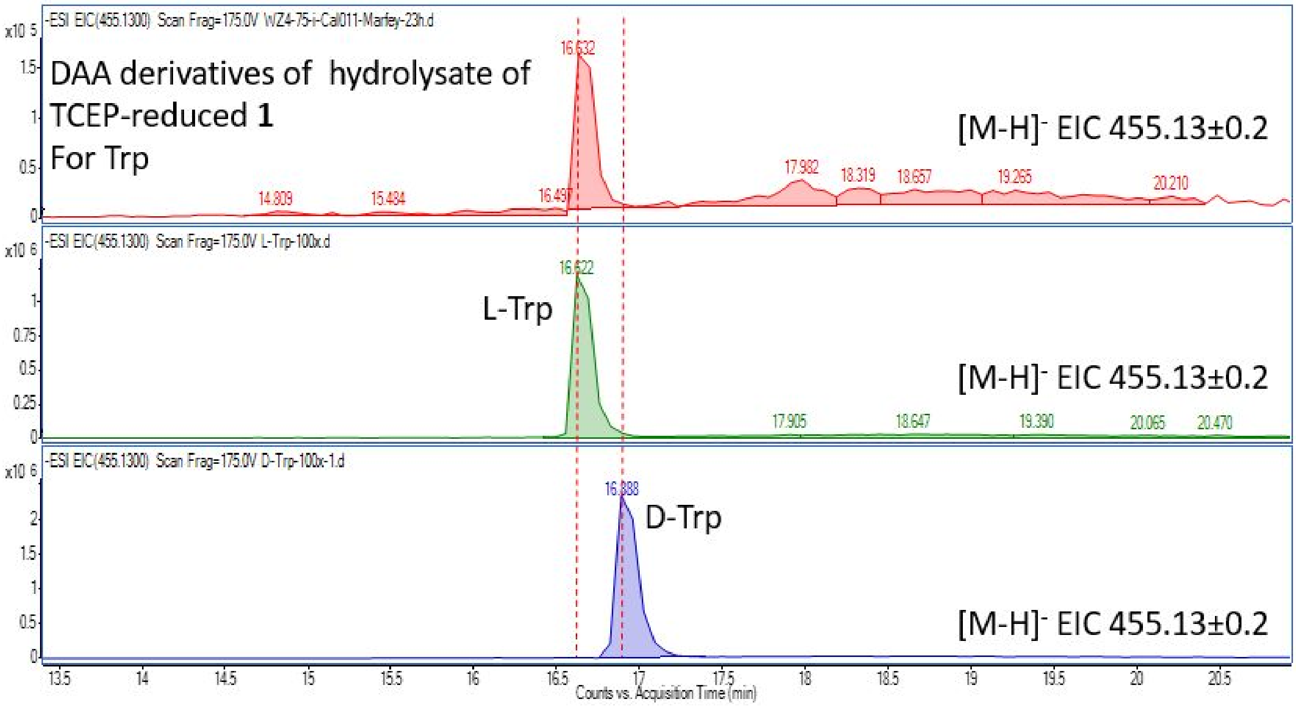

Figure S19. Marfey's analysis to determine the absolute configuration of the Trp residue in TCEP-reduced **1**. EICs demonstrating the retention time of the DAA-derivatized Trp residue resulting from the acid hydrolysis of TCEP-reduced **1** (top), retention time of DAA-derivatized standard of L-Trp (middle), and the retention time of the similarly derivatized standard of D-Trp (bottom). Separation was achieved using the Agilent Poroshell EC-C18 (100×4.6 mm, 2.7 μm) column. Mass spectrometry data were acquired in the negative ionization mode.

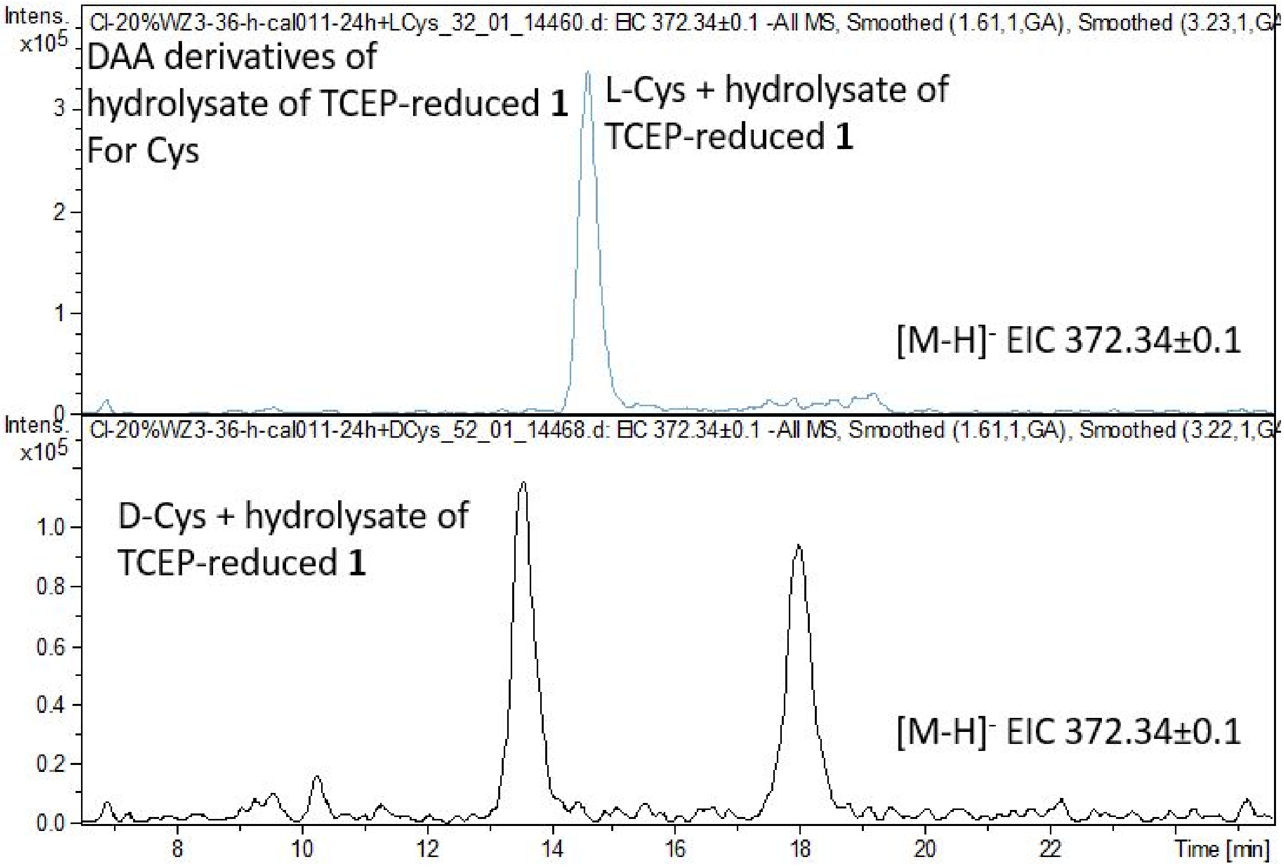

Figure S20. Marfey's analysis to determine the absolute configuration of the Cys residue in TCEP-reduced **1**. EICs demonstrating retention time of DAA-derivatized standard for L-Cys spiked with the derivatized acid hydrolysate of TCEP-reduced **1** (top), and DAA-derivatized standard for D-Cys spiked with the derivatized acid hydrolysate of TCEP-reduced **1** (bottom). By retention time matching, the Cys residue in TCEP-reduced **1** was determined to be L-Cys. Chromatographic separation was achieved using the Cosmosil Cholester (250×4.6 mm, 5 µm) column. Mass spectrometry data were acquired in the negative ionization mode.

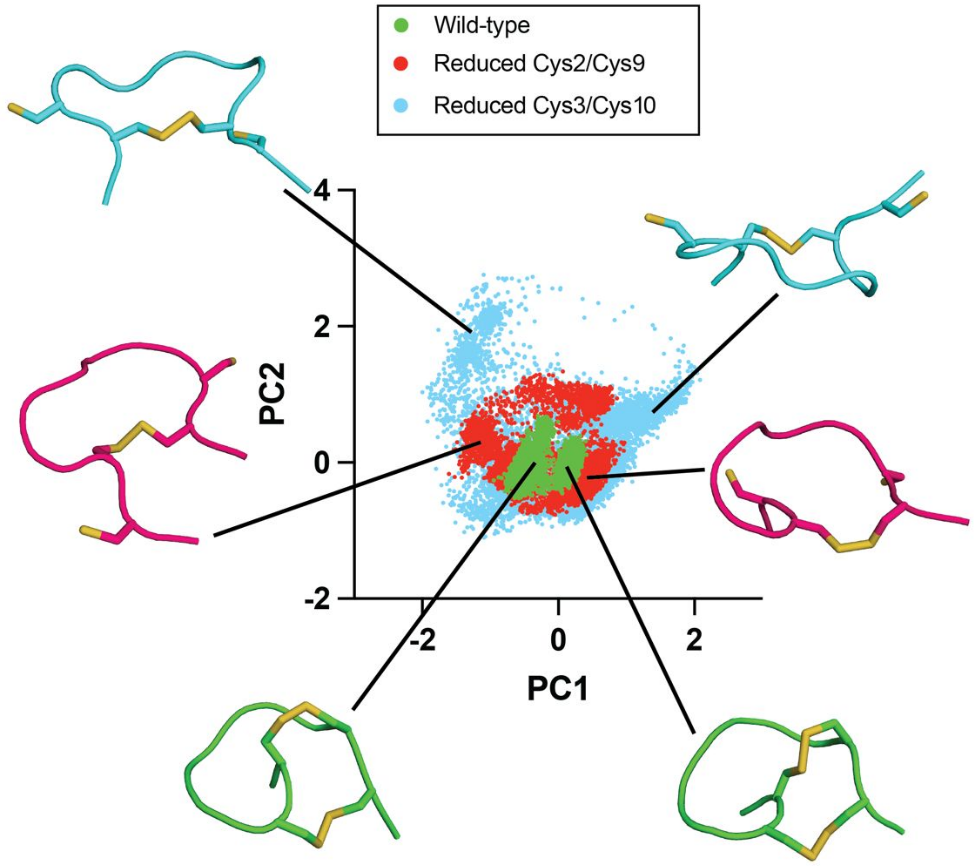

Figure S21. Representative MD simulation derived principal component analysis (PCA) to study the folding dynamics of oxidized and reduced halichondamide A (**1**). Graphs are prepared form the collective motion of oxidized (green) and reduced halichondamide A (**1**, red or cyan) using projections of MD trajectories on two eigenvectors corresponding to the first two principal components. Example conformations are shown to represent different conformations observed in the simulations.

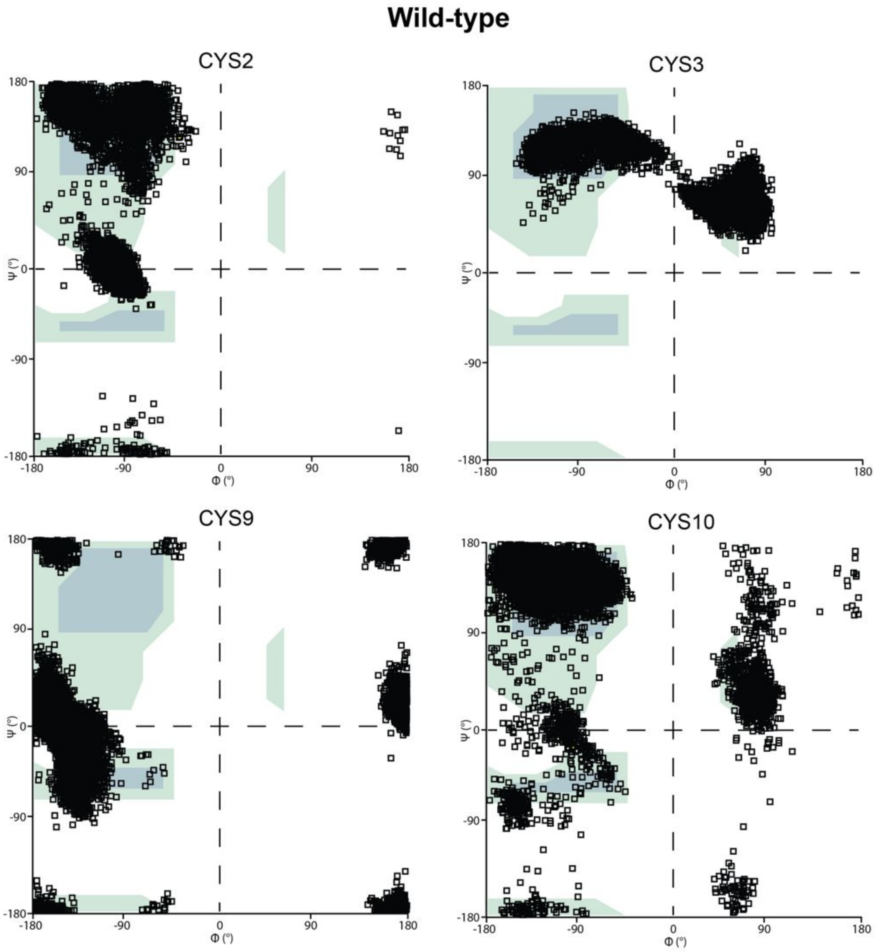

Figure S22. Representative Ramachandran plots of each Cys residue of wild-type (oxidized) halichondamide A (**1**) throughout MD simulations.

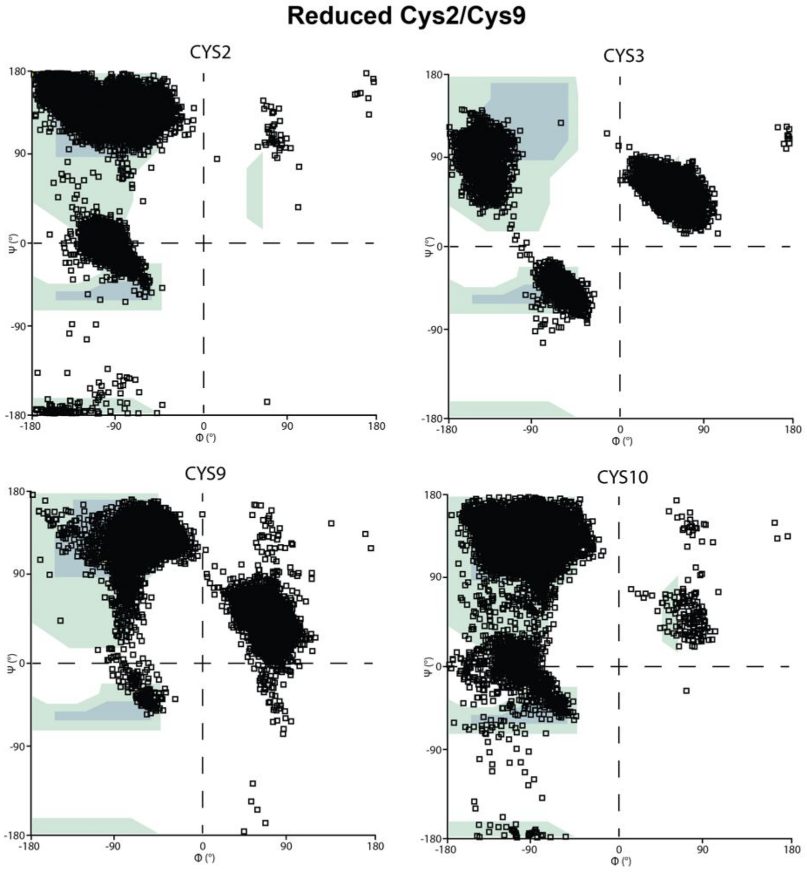

Figure S23. Representative Ramachandran plots of each Cys residue of reduced (Cys2/Cys9 bond) halichondamide A (**1**) throughout MD simulations.

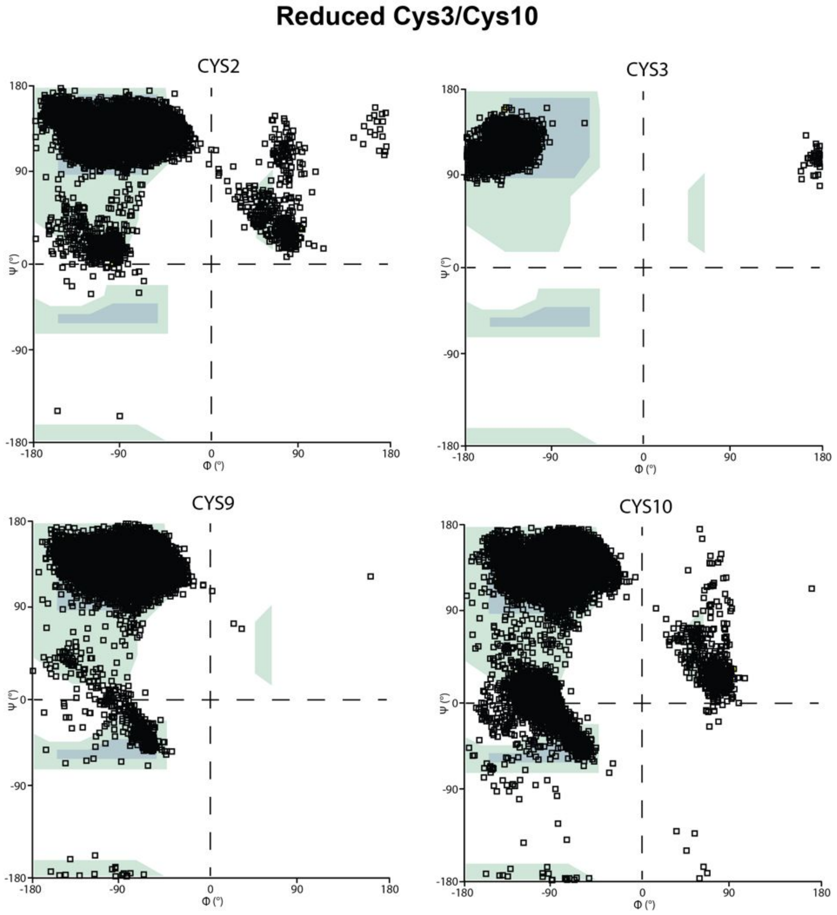

Figure S24. Representative Ramachandran plots of each Cys residue of reduced (Cys3/Cys10 bond) halichondamide A (**1**) throughout MD simulations.

SUPPLEMENTARY REFERENCES

1. Herrmann, T.; Güntert, P.; Wüthrich, K. Protein NMR Structure Determination with Automated NOE Assignment Using the New Software CANDID and the Torsion Angle Dynamics Algorithm DYANA. *J. Mol. Biol.* **2002**, *319*, 209–227.
2. Yadava, S. K.; Basu, S. M.; Valsalakumari, R.; Chauhan, M.; Singhania, M.; Giri, J. Curcumin-Loaded Nanostructure Hybrid Lipid Capsules for Co-eradication of Breast Cancer and Cancer Stem Cells with Enhanced Anticancer Efficacy. *ACS Appl. Bio Mater.* **2020**, *3*, 6811–6822.
3. Jo, S.; Kim, T.; Iyer, V. G.; Im, W. CHARMM-GUI: a Web-based Graphical User Interface for CHARMM. *J. Comput. Chem.* **2008**, *29*, 1859–1865.
4. Van Der Spoel, D.; Lindahl, E.; Hess, B.; Groenhof, G.; Mark, A. E.; Berendsen, H. J. C. GROMACS: Fast, Flexible, and Free. *J. Comput. Chem.* **2005**, *26*, 1701–1718.
5. Humphrey, W.; Dalke, A.; Schulten, K. VMD: Visual Molecular Dynamics. *J. Mol. Graphics* **1996**, *14*, 33–38.
6. Carstens, B. B.; Rosengren, K. J.; Gunasekera, S.; Schempp, S.; Bohlin, L.; Dahlstrom, M.; Clark, R. J.; Goransson, U. Isolation, Characterization, and Synthesis of the Barrettides: Disulfide-Containing Peptides from the Marine Sponge *Geodia barretti*. *J. Nat. Prod.* **2015**, *78*, 1886–1893.
7. Steffen, K.; Laborde, Q.; Gunasekera, S.; Payne, C. D.; Rosengren, K. J.; Riesgo, A.; Goransson, U.; Cardenas, P. Barrettides: a Peptide Family Specifically Produced by the Deep-Sea Sponge *Geodia barretti*. *J. Nat. Prod.* **2021**, *84*, 3138–3146.
8. Li, H.; Bowling, J. J.; Fronczek, F. R.; Hong, J.; Jabba, S. V.; Murray, T. F.; Ha, N.-C.; Hamann, M. T.; Jung, J. H. Asteropsin A: an Unusual Cystine-crosslinked Peptide from Porifera Enhances Neuronal Ca^{2+} Influx. *Biochim. Biophys. Acta, Gen. Subj.* **2013**, *1830*, 2591–2599.
9. Li, H.; Bowling, J. J.; Su, M.; Hong, J.; Lee, B.-J.; Hamann, M. T.; Jung, J. H. Asteropsins B–D, Sponge-derived Knottins with Potential Utility as a Novel Scaffold for Oral Peptide Drugs. *Biochim. Biophys. Acta, Gen. Subj.* **2014**, *1840*, 977–984.
10. Li, H.; Su, M.; Hamann, M. T.; Bowling, J. J.; Kim, H. S.; Jung, J. H. Solution Structure of a Sponge-Derived Cystine Knot Peptide and Its Notable Stability. *J. Nat. Prod.* **2014,** *77*, 304–310.

11. Su, M.; Li, H.; Wang, H.; Kim, E. L.; Kim, H. S.; Kim, E.-H.; Lee, J.; Jung, J. H. Stable and Biocompatible Cystine Knot Peptides from the Marine Sponge *Asteropus* sp. *Bioorg. Med. Chem.* **2016**, *24*, 2979–2987.
12. Takada, K.; Hamada, T.; Hirota, H.; Nakao, Y.; Matsunaga, S.; van Soest, R. W.; Fusetani, N. Asteropine A, a Sialidase-inhibiting Conotoxin-like Peptide from the Marine Sponge *Asteropus Simplex*. *Chem. Biol.* **2006**, *13*, 569–574.
13. Williams, D. E.; Austin, P.; Diaz-Marrero, A. R.; Soest, R. V.; Matainaho, T.; Roskelley, C. D.; Roberge, M.; Andersen, R. J. Neopetrosiamides, Peptides from the Marine Sponge *Neopetrosia* sp. that Inhibit Amoeboid Invasion by Human Tumor Cells. *Org. Lett.* **2005**, *7*, 4173–4176.
14. Liu, H.; Boudreau, M. A.; Zheng, J.; Whittal, R. M.; Austin, P.; Roskelley, C. D.; Roberge, M.; Andersen, R. J.; Vederas, J. C. Chemical Synthesis and Biological Activity of the Neopetrosiamides and their Analogues: Revision of Disulfide Bond Connectivity. *J. Am. Chem. Soc.* **2010**, *132*, 1486–1487.
15. Woo, J. K.; Jeon, J. E.; Kim, C. K.; Sim, C. J.; Oh, D. C.; Oh, K. B.; Shin, J. Gombamide A, a Cyclic Thiopeptide from the Sponge *Clathria Gombawuiensis*. *J. Nat. Prod.* **2013**, *76*, 1380–1383.
